# Metabolic Stress Shifts the Function of K_Ca_3.1 Channels in Pancreatic Beta Cells From a Regulator of Membrane Potential to a Proapoptotic Factor

**DOI:** 10.1002/ardp.70344

**Published:** 2026-10-09

**Authors:** Jurek Hense, Lena Beuke, Christina Kick, Bernhard Wünsch, Martina Düfer

**Affiliations:** ^1^ Institute of Pharmaceutical and Medicinal Chemistry, Department of Pharmacology University of Münster Münster Germany; ^2^ Institute of Pharmaceutical and Medicinal Chemistry University of Münster Münster Germany

**Keywords:** electrophysiology, glucolipotoxicity, KCa3.1 channel, NMDA receptor, pancreatic islet

## Abstract

Metabolic stress increases the whole‐cell current through K_Ca_3.1 channels in mouse pancreatic islet cells. The study aimed to analyze the network between ion channels mediating Ca^2+^ influx for K_Ca_3.1 channel activation and stress‐related changes. The physiologically important K_slow_ current of beta cells was not altered in total but the contribution of K_Ca_3.1 current was reduced after culture in the presence of high glucose and palmitate concentrations. Electrical activity was increased by blocking K_Ca_3.1 channels with senicapoc in islets cultured under standard conditions but not after metabolic stress. Of note, the functional coupling of K_Ca_3.1 to l‐type Ca^2+^ channels was severely reduced by metabolic stress, whereas the coupling to Ca^2+^‐influx via NMDA receptors increased. Mitochondrial membrane potential of beta cells was not affected by senicapoc but by a mitochondria‐targeted derivative. Inhibition of K_Ca_3.1 channels by senicapoc or maurotoxin partly protected from apoptosis. In conclusion, the data indicate a shift in the interplay of Ca^2+^‐conducting ion channels and the plasma membrane‐localized K_Ca_3.1 channel. K_Ca_3.1 channels changed from an important regulator of membrane potential oscillations to a dysfunctional and proapoptotic factor. This switch might be involved in the decline of islet cell mass and function during progression of type 2 diabetes mellitus.

## Graphical abstract

K_ATP_ and K_Ca_3.1 channels are the main components of the regulatory K^+^ current “K_slow_” of pancreatic beta cells. In a diabetogenic environment, the K_Ca_3.1 component of K_slow_ decreases, and the channel is dysregulated. A targeted pharmacological approach reveals that K_Ca_3.1 channels are functional in mitochondria. Only inhibition of the plasma membrane channel protects against the pro‐apoptotic influence K_Ca_3.1 channels gain under metabolic stress.

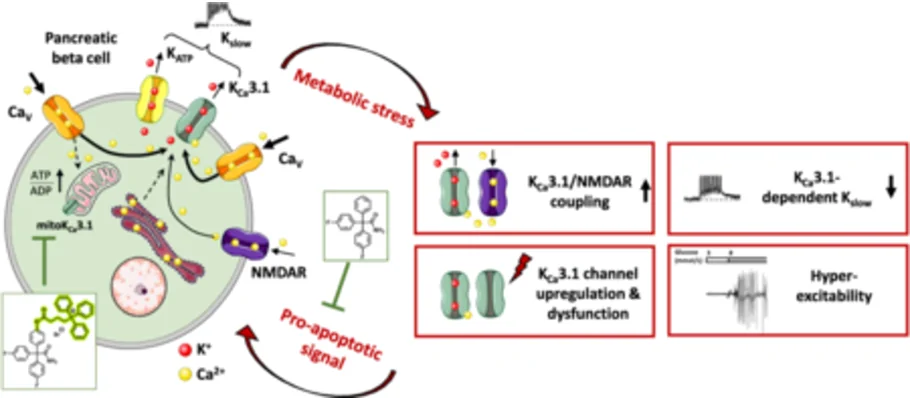

**Abbreviations**
Ca_V_ channels: voltage‐activated Ca^2+^ channels
[Ca^2+^]_i_
: intracellular Ca^2+^ concentration
ER: endoplasmic reticulum
GLT: glucolipotoxicity
K_V_ channels: voltage‐activated K^+^ channels
K_ATP_ channels: ATP‐dependent K^+^ channels
K_Ca_ channels: Ca^2+^‐activated K^+^ channels
NMDAR: N‐methyl‐d‐aspartate receptors
NLRP3: NLR family pyrin domain containing 3
ROS: reactive oxygen species

## 1 Introduction

Several K^+^ channels are expressed in pancreatic beta cells and are essential for the proper function of this cell type. The ATP‐dependent potassium channel (K_ATP_) and voltage‐dependent potassium channels are important for the regulation of membrane potential and therefore main modulators of insulin secretion [1, 2, 3]. K_ATP_ channels, which are well‐known drug targets, are essential for the maintenance of the resting membrane potential and repolarization of the cell after bursts of action potentials, while K_V_2.1 channels are mainly contributing to the repolarization phase of a single action potential [2, 3, 4]. In addition to these K^+^ channels, Ca^2+^‐activated K^+^ channels are expressed in pancreatic beta cells and are known to participate in the modulation of electrical activity [5, 6, 7]. Their significance for proper cell function was demonstrated for excitable and non‐excitable cells [8, 9, 10]. K_Ca_2.1‐2.3 and K_Ca_3.1 channels are activated solely due to a rise in intracellular calcium concentration ([Ca^2+^]_i_) to a submicromolar range (100–350 nM) and operate independently of changes in membrane voltage, contrary to K_Ca_1.1 channels, whose activation is dependent on [Ca^2+^]_i_ and membrane voltage changes [11, 12]. The rise in [Ca^2+^]_i_ needed for the activation of K_Ca_3.1 channels in pancreatic beta cells can originate from extracellular Ca^2+^ sources, mainly voltage‐gated Ca^2+^ channels, as well as from the endoplasmic reticulum (ER) [13, 14]. The K_Ca_3.1 channel is contributing to the so‐called K_slow_ current of the islets, which is a slowly activating and slowly inactivating K^+^ current, elicited by the Ca^2+^ influx that occurs during the rhythmical burst phases of electrical activity [5, 13]. While this physiological function is well‐established, the role of K_Ca_3.1 channels during progression of diabetes is sparsely investigated. K_Ca_3.1 channel expression is upregulated due to metabolic or inflammatory stress [15, 16], and a beneficial effect of blocking K_Ca_3.1 channels on glucose tolerance was shown in diabetic mice [15]. Furthermore, cytokines were shown to reduce the K_slow_ current, but the role of K_Ca_3.1 channels was not addressed in that study [17]. The aim of our investigation is to further elucidate the pathophysiological role of K_Ca_3.1 channels for the progression of beta cell failure, with a focus on the characterization of electrophysiological changes in the ion channel network and the potential of ion channel modulators.

## 2 Results

### 2.1 Metabolic Stress Altered the Dependence of K_Ca_3.1 Channel Activation on l‐Type Calcium Channels

We previously reported an increase in K_Ca_3.1 expression that was accompanied by a rise in the senicapoc‐sensitive proportion of the whole‐cell K^+^ current in mouse beta cells exposed to stress conditions [16]. To elucidate which source of Ca^2+^ is coupled to the elevation in K_Ca_3.1 current, whole‐cell currents were measured in the absence or presence of the l‐type Ca^2+^ channel inhibitor nifedipine (5 µmol/L) in islet cells after cultivation in standard culture medium or in medium supplemented with 25 mmol/L glucose and 100 µmol/L BSA‐bound palmitate for 48 h. Currents were elicited by a voltage‐step protocol and evaluated between −60 and 30 mV to detect changes in the physiologically relevant ranges. In non‐stressed beta cells, nifedipine reduced the whole‐cell K^+^ current as expected, confirming that K^+^ current dependent on Ca^2+^ influx via l‐type Ca^2+^ channels could be measured with this protocol (Figure 1A, green *vs*. black curve). The effect of the selective K_Ca_3.1 channel blocker senicapoc (1 µmol/L) was lower than the one of nifedipine (Figure 1A, green *vs*. blue curve), and the combination (red curve in Figure 1A) had no significant influence compared with nifedipine alone. These characteristics changed after cultivation of the islet cells in glucolipotoxic medium. In stressed beta cells, the whole‐cell K^+^ current was reduced to the same extent by nifedipine and senicapoc, respectively (compare the green and blue curves in Figure 1B). Acute application of senicapoc in the presence of nifedipine revealed that beta cells pre‐treated with glucolipotoxicity were more responsive to the K_Ca_3.1 channel inhibitor under conditions where l‐type Ca^2+^ channels were blocked than the corresponding controls (Figure 1A,B; compare the respective “delta” between green and red curves). These data reveal that metabolic stress decreases the dependence of K_Ca_3.1 channels on Ca^2+^ influx through l‐type Ca^2+^ channels.

**Figure 1:**
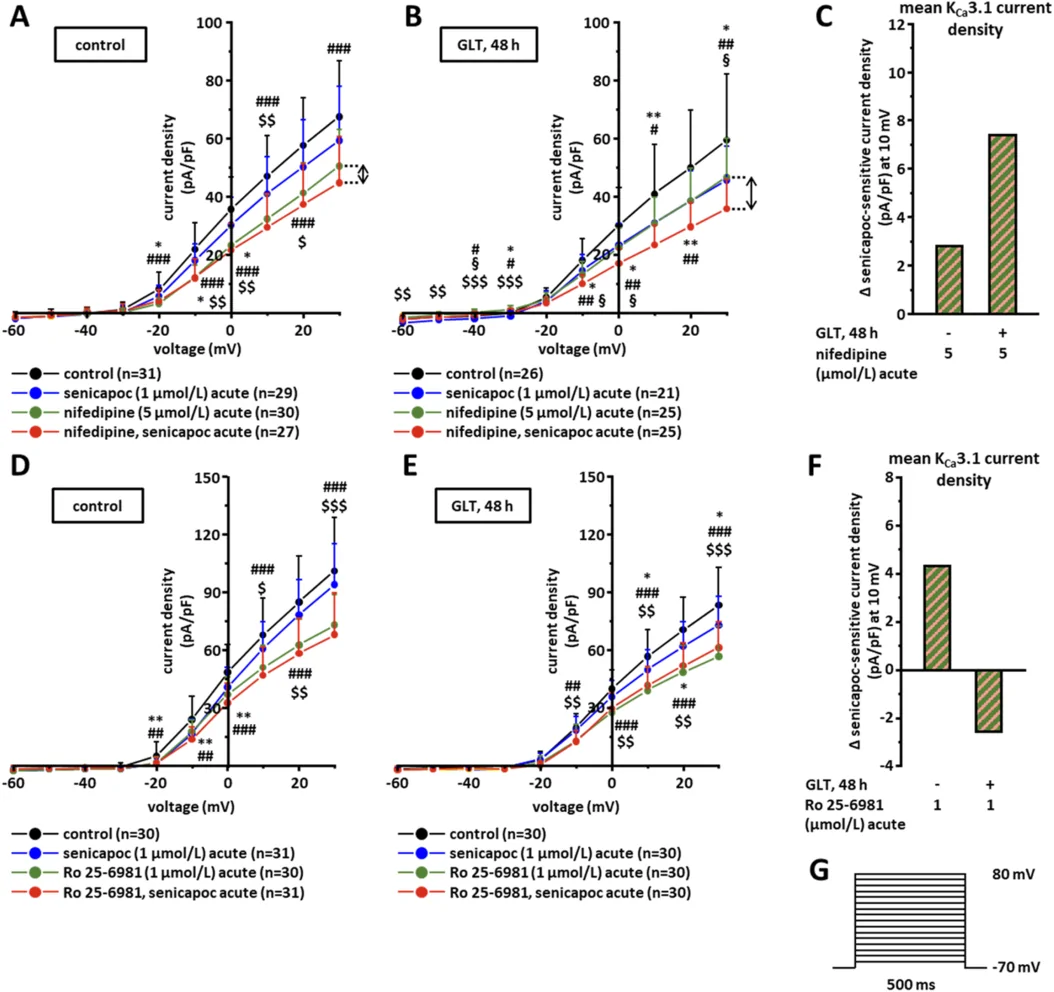
Whole‐cell K^+^ currents were measured in cells treated with control conditions (A) or glucolipotoxicity (GLT) (B). (A) Currents were measured either in the presence of senicapoc (1 µmol/L), nifedipine (5 µmol/L), a combination of both or without the inhibitors. Nifedipine blocked the current to a greater extent than senicapoc in control cells indicating inhibition of Ca^2+^‐dependent K^+^ currents. Senicapoc blocked a small additional current in the presence of nifedipine (green vs. red). (B) In GLT‐pretreated cells, senicapoc and nifedipine inhibited the current to a similar extent but senicapoc exerted a larger effect in the presence of nifedipine than it did in control cells. (C) Mean K_Ca_3.1 currents at the voltage step to 10 mV. K_Ca_3.1 current was less reduced in the presence of nifedipine in metabolically stressed cells *versus* control. (D/E) Similar experiment as in A/B but performed with Ro 25‐6981 (1 µmol/L) instead of nifedipine. Ro 25‐6981 blocked the K^+^ current in control cells (e.g. 25% at 10 mV and 28% at 30 mV) and glucolipotoxicity‐treated cells (e.g., 31% at 10 mV and 32% at 30 mV) (black vs. green). In the presence of Ro 25‐6981, senicapoc merely reduced the current further in control cells but not in GLT‐treated cells (green vs. red). (F) Mean K_Ca_3.1 currents at the voltage step to 10 mV. It was only detectable in control cells but not in glucolipotoxicity pre‐treated cells. (G) Voltage protocol. Only the steps up to 30 mV are presented in A, B, D and E. The whole range up to 80 mV is shown in Supporting Information S1: Figure 3. Male/female: 1/2 (A,B), 1/3 (D,E) preparations. *p < 0.05, **p < 0.01 for control *versus* senicapoc; ##*p* < 0.01, ###*p* < 0.001 for control versus nifedipine or Ro 25‐6981; $*p* < 0.05, $$*p* < 0.01, $$$*p* < 0.001 for senicapoc *versus* nifedipine or Ro 25‐6981; §*p* < 0.05 for nifedipine *versus* nifedipine + senicapoc.

The diagram in Figure 1C illustrates the differences in K_Ca_3.1 current (i.e., senicapoc‐sensitive current) between the I/V curves for the voltage step to 10 mV to exemplarily visualize the glucolipotoxicity‐mediated changes. In the presence of nifedipine, the mean K_Ca_3.1 current that could be blocked by senicapoc amounts to ~2.8 pA/pF in control cells. Glucolipotoxicity reduced the influence of nifedipine, and with ~7.4 pA/pF more than twice as much of the K^+^ current was inhibited by senicapoc.

Since nifedipine can block K_Ca_3.1 channels and K_V_2.1 channels, when the channels are overexpressed in cell lines [18, 19], an additional series of experiments was performed with diltiazem for which these off‐target effects are not described. Diltiazem blocked the whole‐cell current to the same extent as nifedipine (Supporting Information S1: Figure 1A), indicating that putative off‐target effects were not relevant for our protocol in primary beta cells. It was also excluded that 1 µmol/L senicapoc inhibited K_ATP_ channels, which play an outstanding role for the regulation of electrical activity of beta cells (Supporting Information S1: Figure 1B).

### 2.2 Metabolic Stress Increased the Coupling Between Ca^2+^ Influx via NMDA Receptors and K_Ca_3.1 Channels

As the interaction between K_Ca_3.1 channels and Ca^2+^ influx through l‐type Ca^2+^ channels was impaired in response to metabolic stress, we investigated the relevance of other Ca^2+^ sources for channel activation. NMDA receptors (NMDARs) are known for their potential to stimulate K_Ca_ channels in beta‐cells and for their negative influence on islets exposed to stress conditions [16, 20, 21]. To investigate putative glucolipotoxicity‐induced alterations, whole‐cell K^+^ currents were measured in the presence or absence of the NMDAR antagonist Ro 25‐6981 (1 µmol/L), which selectively targets NMDAR via the subunit GluN2B. Blocking NMDARs substantially reduced the whole‐cell current in control cells. Senicapoc had a small additional inhibitory influence in the presence of Ro 25‐6981 (Figure 1D, red *vs.* green curve). This additional effect of senicapoc was absent after 48 h of glucolipotoxic culture, indicating an increased dependence of K_Ca_3.1 channel activation on Ca^2+^ originating from GluN2B‐containing NMDARs (Figure 1E). Figure 1F illustrates the difference in senicapoc‐sensitive K^+^ current in the presence of the NMDAR inhibitor in stressed *versus* non‐stressed beta cells at the voltage step to 10 mV expressed as overall change. To rule out any off‐target effects of Ro 25‐6981 on K_v_ channels, the compound was tested in a Ca^2+^‐free bath solution. Under these conditions Ro 25‐6918 had no effect on the K_v_ current (Supporting Information S1: Figure 2).

To further elucidate the interaction between NMDARs and K_Ca_3.1 channels, cells were acutely stimulated with NMDA/glycine (500/10 µmol/L) to open all NMDARs that were activatable after culture in control or glucolipotoxic medium. Current–voltage relationships are shown in Figure 2A. Metabolic stress (48 h) led to an increase in whole‐cell current in response to acute NMDAR activation, which was sensitive to blocking NMDARs (Figure 2A: difference black and blue curve *vs*. difference red and green curve). Figure 2B exemplarily illustrates for the voltage step to 10 mV that the Ro 25‐6981‐sensitive current was doubled in metabolically stressed cells, demonstrating that metabolic stress increases the outward current dependent on Ca^2+^ influx originating from GluN2B‐containing NMDARs. Acute application of senicapoc (1 µmol/L) to metabolically stressed cells in the presence of Ro 25‐6981 (1 µmol/L, orange curve in Figure 2A) had only a slight additional inhibitory effect, further supporting the hypothesis that the influence of NMDAR‐mediated Ca^2+^ influx on K^+^ currents after treatment of the cells with glucolipotoxicity is at least partly due to activation of K_Ca_3.1 channels. Of note, the effect of NMDA/glycine and the inhibitory influence of senicapoc alone on NMDA/glycine‐induced K^+^ currents were not tested in this series of experiments as they had already been investigated previously [16]. Figure 2C displays the overlay of exemplary recordings for beta cells stimulated with NMDA/glycine under the various conditions (of note, the whole range of voltage steps from −10 to 80 mV is illustrated in these traces; see Supporting Information S1: Figure 3 for corresponding I/V curves). In addition to the influence of Ro 25‐6981 on the magnitude of K^+^ currents, the inhibition of GluN2B‐subunit containing NMDARs altered the kinetic of the whole‐cell currents unmasking an inactivating component of the remaining K^+^ current. V_1/2_ was 17 ± 4 mV in the presence of NMDA/glycine and 10 ± 5 mV when Ro 25‐6981 was added (*p* < 0.001), the slope factor changed from K = 21 ± 3 mV to K = 15 ± 3 mV (*p* < 0.001), respectively.

**Figure 2:**
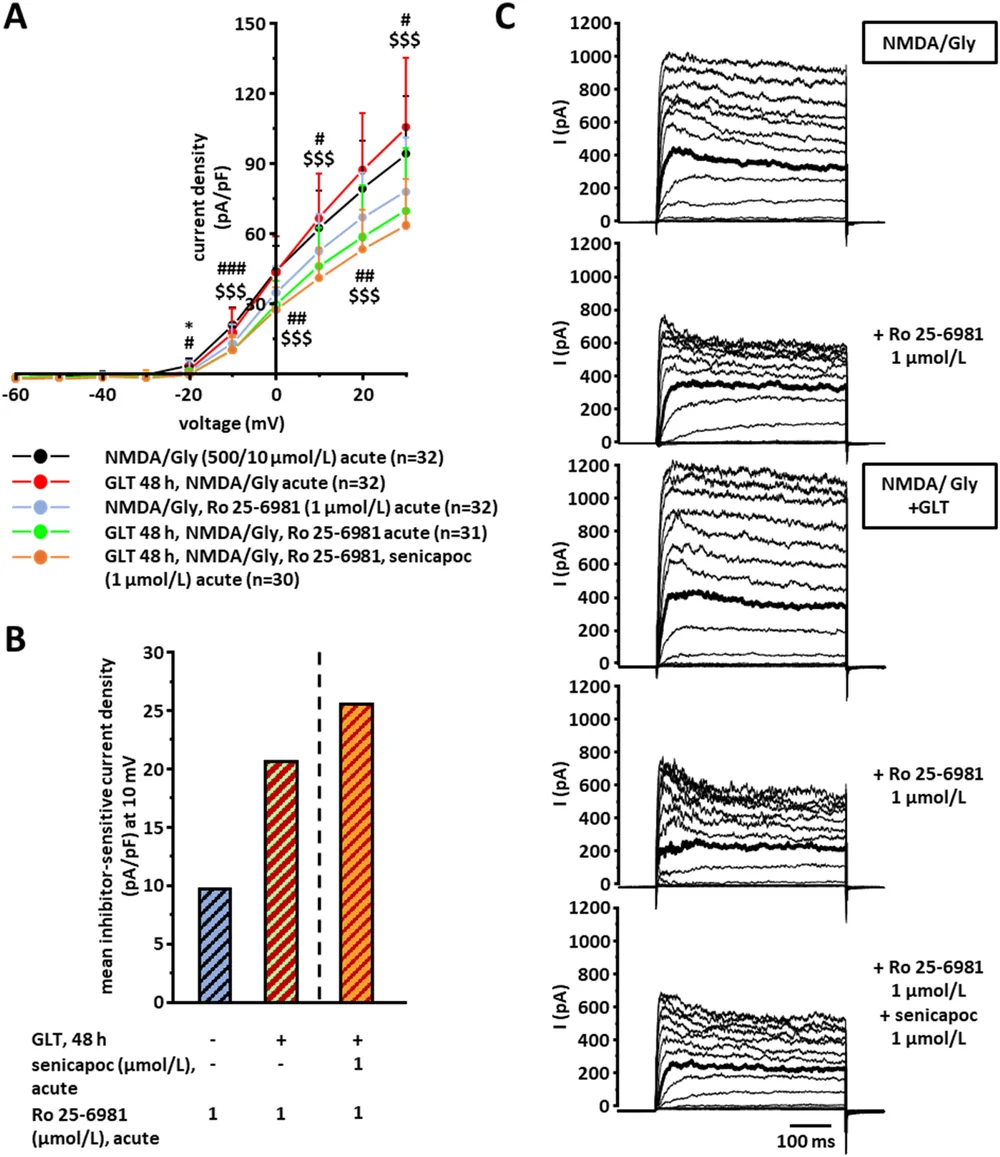
Whole‐cell current voltage relationships were acquired in the presence of NMDA (500 µmol/L) and glycine (10 µmol/L) and indicated inhibitors (1 µmol/L). (A) A subset of cells was treated with glucolipotoxicity (GLT) before. Ro 25‐6981 (1 µmol/L) inhibited K^+^ currents significantly in control cells and GLT‐pretreated cells (black *vs.* blue and red *vs.* green). Senicapoc (1 µmol/L) blocked additional current in GLT‐pretreated cells in the presence of Ro 25‐6981 (male/female: 2/2 preparations). (B) Mean K^+^ currents sensitive to indicated inhibitors at the voltage step to 10 mV. Ro 25‐6981 (1 µmol/L) sensitive current was almost doubled due to GLT preincubation (the difference of blue/black *vs.* difference of green/red) demonstrating increased Ca^2+^‐induced K^+^ current. Senicapoc (1 µmol/L) further increased the blockable current in GLT‐pretreated cells to a small extent (orange/red). (C) Exemplary measurements for every condition shown in (A). Note the high reduction and altered current inactivation at higher voltages by Ro 25‐6981. *p < 0.05 for NMDA/Gly *versus* GLT + NMDA/Gly; #*p* < 0.05, ##*p* < 0.01, ###*p* < 0.001 for NMDA/Gly *versus* NMDA/Gly + Ro25‐698; $$$*p* < 0.001 for GLT + NMDA/Gly *versus* GLT + NMDA/Gly + Ro 25‐6981.

### 2.3 Metabolic Stress Affected the Physiological Function of the K_Ca_3.1 Channels in Pancreatic Islets

As our data show, that the activation of K_Ca_3.1 channels becomes less sensitive to Ca^2+^ influx via l‐type Ca^2+^ channels, we further checked if this alteration had any influence on the regulation of the nutrient‐dependent oscillations in membrane potential that characterize the islets of Langerhans. Physiologically, an accumulation of Ca^2+^ directly activates K_Ca_3.1 channels and indirectly K_ATP_ channels thereby providing a hyperpolarizing K_slow_ current that transiently interrupts electrical activity as mentioned in the introduction. The K_slow_ current was calculated as the difference of the outward current before and directly after the application of a train of voltage ramps from −40 mV to 0 mV (Figure 3G), mimicking a burst of action potentials (Figure 3B, indicated by red arrows) in control *versus* stressed beta cells. Despite the rise of K_Ca_3.1 current measured in the whole‐cell configuration, the K_slow_ current was not altered after glucolipotoxic cell stress (Figure 3A, and exemplary traces in 3B, Supporting Table for sex‐specific evaluation). To test if the lack of any effect is due to a compensatory rise in the contribution of K_ATP_ channels to the K_slow_ current, we performed the same experiment with cells of SUR1^‐/‐^ mice (Figure 3C). As expected, the K_slow_ current of SUR1^‐/‐^ cells *per se* was significantly lower compared with WT cells, but glucolipotoxic cell stress did not alter the K_slow_ current either. Next, we checked to what extent blocking K_Ca_3.1 current is reducing K_slow_ current in metabolically stressed cells compared with control cells. K_slow_ current was measured before and after the application of senicapoc (1 µmol/L), and the relative K_Ca_3.1 fraction of K_slow_ was calculated. In control cells, senicapoc reduced the current to a similar extent as described for another well‐established K_Ca_3.1 inhibitor TRAM‐34 [5]. Surprisingly, the K_slow_ current was only half as sensitive to senicapoc in metabolically stressed cells (Figure 3D). In control cells, senicapoc reduced K_slow_ current by approximately 44% (2.2 ± 1.3 vs. 1.3 ± 1.2 pA/pF, *p* < 0.001, exemplary recording in Figure 3E), while the reduction was 22% in metabolically stressed cells (2.0 ± 0.5 *vs.* 1.5 ± 0.3 pA/pF; *p* < 0.01, exemplary recording in Figure 3F).

**Figure 3:**
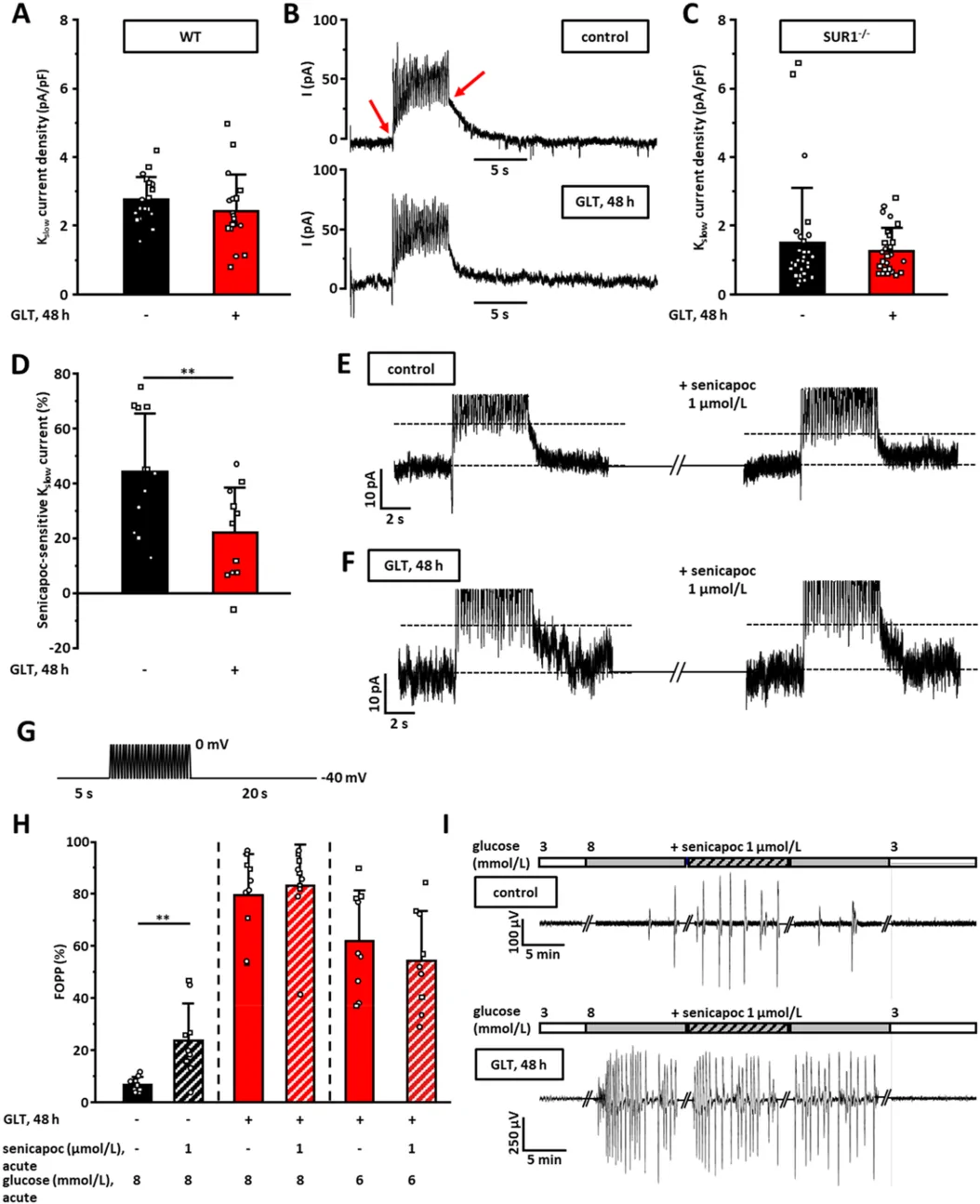
Changes in K_slow_ current of beta cells and electrical activity of islets due to glucolipotoxicity (GLT). (A) The K_slow_ current was measured in the perforated‐patch configuration. K_slow_ was not altered in cells of wildtype animals (WT) pre‐treated with GLT compared with control. (B) Exemplary measurements of K_slow_ current in WT cells. The first red arrow marks the basal current right before application of the train of voltage ramps, and the second arrow marks the current directly after the end of the voltage train. Difference of both values (mean of 500 ms before and mean of 200 ms after the train of action potentials) was calculated as K_slow_ current. (C) K_slow_ current was also measured in SUR1^‐/‐^ cells. GLT did not alter K_slow_ current in SUR1^–/–^ cells. (D) K_slow_ current was measured before and after the application of senicapoc (1 µmol/L), and the senicapoc‐sensitive current was determined by subtraction of both currents. Measurements were performed with control cells and GLT‐pre‐treated cells. The senicapoc‐sensitive K_slow_ current was significantly reduced in metabolically stressed cells. (E) Exemplary traces of K_slow_ currents in a control cell before and after the application of senicapoc. Note the clear reduction in K_slow_ current. (F) Exemplary traces of K_slow_ currents in cells treated with GLT for 48 h before and after the acute application of senicapoc. (G) Protocol used for induction of K_slow_ current. (H) Electrical activity of islets was determined with a micorelectrode array. Islets were stimulated with 8 mmol/L of glucose. Senicapoc (1 µmol/L) increased electrical activity of control islets significantly (left part, black bars). Islets stressed with glucolipotoxicity showed high basal electrical activity when stimulated with 8 mmol/L and 6 mmol/L of glucose. Senicapoc (1 µmol/L) did not alter electrical activity of these islets (middle and right part). (I) Exemplary measurements of electrical activity of islets. Islets were electrically inactive in the presence of 3 mmol/L glucose. Elevating glucose to 8 mmol/L increased electrical activity. The addition of senicapoc further elevated the FOPP in control islets (upper trace) but not in islets stressed with glucolipotoxicity (lower trace). Islets were electrically silent again with 3 mmol/L glucose. Squares represent data points acquired from male mice; circles show data points from female mice. Male/Female: 3/2 (A, C), 2/1 (D, control), 3/1 (D, GLT), 2‐3/3 (H) preparations. **p < 0.01 as indicated.

### 2.4 Metabolic Stress Abolished the Effect of Senicapoc on Electrical Activity of Islets of Langerhans

The reduced contribution of K_Ca_3.1 channels to K_slow_ points to a decreased functional influence of the K_Ca_3.1 current. Therefore, we investigated whether the effect of senicapoc on membrane potential of whole islets of Langerhans was also decreased by metabolic stress. Isolated islets were cultured in glucolipotoxic or standard medium for 48 h and placed on a microelectrode‐array (MEA) chip thereafter. After silencing electrical activity in low glucose (3 mmol/L), islets were stimulated with 8 mmol/L glucose for 10 min, and senicapoc (1 µmol/L) was added for another 10 min. For each condition, the fraction of plateau phase with electrical activity (FOPP) was determined. Control islets showed electrical activity with a FOPP below 50% that was increased approximately 3.5‐fold by senicapoc (Figure 3H, left). By contrast, metabolically stressed islets showed already high electrical activity when stimulated with 8 mmol/L glucose while blocking K_Ca_3.1 channels did not further increase this activity (Figure 3H, middle, exemplary recording in Figure 3I, Supporting Table for sex‐dependent evaluation). To exclude that the glucose‐mediated stimulation was already too strong to see any elevation by senicapoc, we reduced the glucose concentration for acute stimulation after glucolipotoxic culture to 6 mmol/L. Electrical activity elicited by 6 mmol/L glucose was approximately 20% lower compared with that by 8 mmol/L of glucose (80 ± 15% *vs.* 61 ± 18% FOPP; p < 0.05), but blocking K_Ca_3.1 channels was still ineffective (Figure 3H, last two bars from right). Extending the glucolipotoxic culture from 48 h up to 13 days resulted in a higher percentage of islets not responding to acute stimulation by 8 mmol/L glucose (days 5‐10: 14%, *n* = 21, days 12 + 13: 47%, *n *= 19). Glucose‐responsive islets were still hyperexcitable (FOPP > 95%, *n* = 10 islets). Senicapoc was ineffective irrespective of the glucose‐responsiveness (not shown). In summary, metabolic stress drastically reduced the hyperpolarizing property of K_Ca_3.1 channels in single cells and islets.

### 2.5 Ca^2+^ Influx through NMDARs Did Not Influence K_Slow_ in Islet Cells

As outlined above, we observed an increased contribution of NMDA/glycine‐mediated activation of NMDARs to the overall whole‐cell current through K^+^ channels in islet cells stressed by glucolipotoxicity. Based on this, the question arises, whether K_slow_ becomes sensitive to NMDAR‐mediated Ca^2+^ influx. To address this issue, we compared the influence of l‐type Ca^2+^ channel inhibition, NMDAR inhibition, and direct activation on K_slow_ in metabolically intact islet cells cultured under standard conditions or in glucolipotoxic medium. As described above, K_slow_ was induced by a train of voltage ramps (Figure 3G). The fraction of K_slow_ sensitive to nifedipine (5 µmol/L) was similar in non‐stressed and metabolically stressed islet cells (Figure 4A). Overall, blocking GluN2B‐containing NMDARs by Ro 25‐6981 (1 µmol/L) had no influence on K_slow_ in cells after 48‐h culture in glucolipotoxic medium (Figure 4B, right bar). Remarkably, in three of the eight cells evaluated, K_slow_ rose in response to the NMDAR inhibitor. This unexpected effect was even more visible in the controls, where K_slow_ was elevated by Ro 25‐6981 in six out of eight islet cells (Figure 4B, left bar). Acute application of NMDA/glycine (500/10 µmol/L) neither resulted in elevated K_slow_ current in islet cells cultivated in standard medium nor in cells exposed to metabolic stress before (Figure 4C).

**Figure 4:**
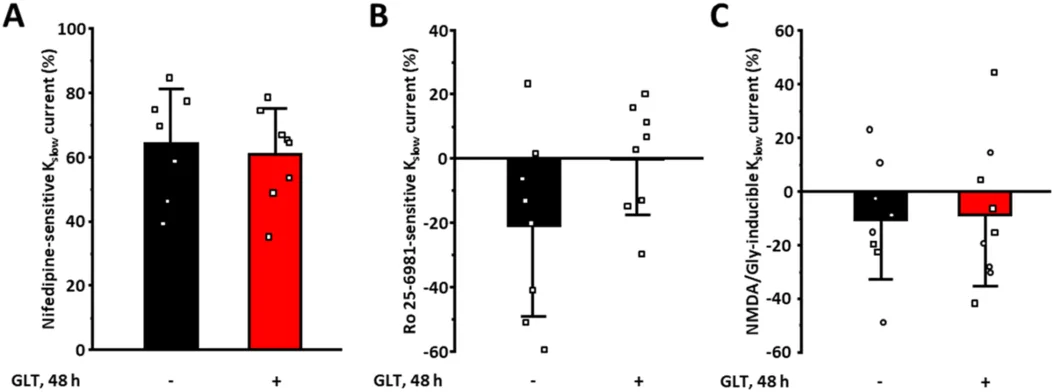
Ca^2+^‐source dependency of K_slow_ current. Same protocol as illustrated in Figure 3G. (A) Fraction of nifedipine‐sensitive K_slow_ current. K_slow_ current was measured in cells cultured under control conditions or in cells challenged with glucolipotoxicity (GLT) for 48 h. To determine the nifedipine‐sensitive fraction, the current was measured before and after the application of nifedipine (5 µmol/L). No change in the nifedipine sensitivity due to glucolipotoxicity was detected. (B) Fraction of Ro 25‐6981 sensitive K_slow_ current. Similiar experiment as in A, but Ro 25‐6981 (1 µmol/L) was used to obtain the GluN2B‐dependent fraction of the K_slow_ current. No statistically significant changes were detected when cells were treated with GLT for 48 h before. Note that Ro 25‐6981 tended to increase (negative sensitivity) K_slow_ current in control cells. (C) NMDA/Gly‐inducible K_slow_ current. NMDA (500 µmol/L) and glycine (10 µmol/L) were applied after 48‐h cultivation in control or GLT medium. Contrary to the expectations, NMDA/Gly tended to decrease (negative inducibility) K_slow_ current to a small extent. No changes due to glucolipotoxicity were detected. Squares represent data points acquired from male mice; circles show data points from female mice. Male/Female: A and B: 3/0, C: 2/2 preparations.

### 2.6 Senicapoc Protected From Apoptosis Induced by Metabolic Stress via Plasma Membrane K_Ca_3.1 Channels

It is known that in vivo treatment of db/db mice with a K_Ca_3.1 channel blocker protects from loss of islet cell mass [15] and we previously reported that K_Ca_3.1 channel inhibition by senicapoc has a direct anti‐apoptotic effect on pancreatic islet cells [16]. As there is evidence that K_Ca_3.1 channels are not only present in the plasma membrane but also in the mitochondria of islet cells [11], we investigated whether mitochondrial membrane potential was affected by the cell‐permeable blocker senicapoc. Figure 5A shows that senicapoc (1 and 10 µmol/L) did not influence the mitochondrial membrane potential of mouse beta cells when added acutely. Even the high concentration of 30 µmol/L applied for 18 h was ineffective (Figure 5B). By contrast, the mitochondria‐targeted senicapoc derivative WMS‐98 03 (1.5 µmol/L, see Experimental section 5.3 for synthesis), which was used as a tool to discriminate between channel inhibition at the plasma membrane or mitochondria, depolarized the mitochondrial membrane potential within minutes (Figure 5C), ensuring that inhibition of mitochondrial K_Ca_3.1 channels is detectable in beta cells. To exclude that the positively charged moiety of WMS‐98 03 influences mitochondrial membrane potential, the cleavage product propyltriphenylphosphonium was tested separately. The compound did not affect the mitochondrial membrane potential and reactivity to glucose (Figure 5D). Finally, we used the membrane‐impermeable K_Ca_3.1 channel blocker maurotoxin and compared its effects on islet cell death to the cell‐permeable inhibitor senicapoc. Isolated islet cells were challenged with 33 mmol/L glucose and 500 µmol/L palmitate for 7 days. This established model led to a moderate increase in apoptosis (Figure 5E,F, first two bars from left). Supplementation of the glucolipotoxic culture medium with senicapoc (1 µmol/L) or maurotoxin (20 nmol/L) partly protected from this damage. After normalizing the data to the rate of glucolipotoxicity‐mediated apoptosis, it became obvious that both K_Ca_3.1 inhibitors reduced the rate of apoptosis to a similar extent (63 ± 20 vs. 72 ± 11%, not statistically different), while the influence of senicapoc seems to be more variable (Figure 5F, hatched red bars). Based on these data, K_Ca_3.1 channels located in the plasma membrane are considered as the main mechanism for the partial reduction of glucoliptotoxicity‐induced islet cell death by K_Ca_3.1 channel inhibition.

**Figure 5:**
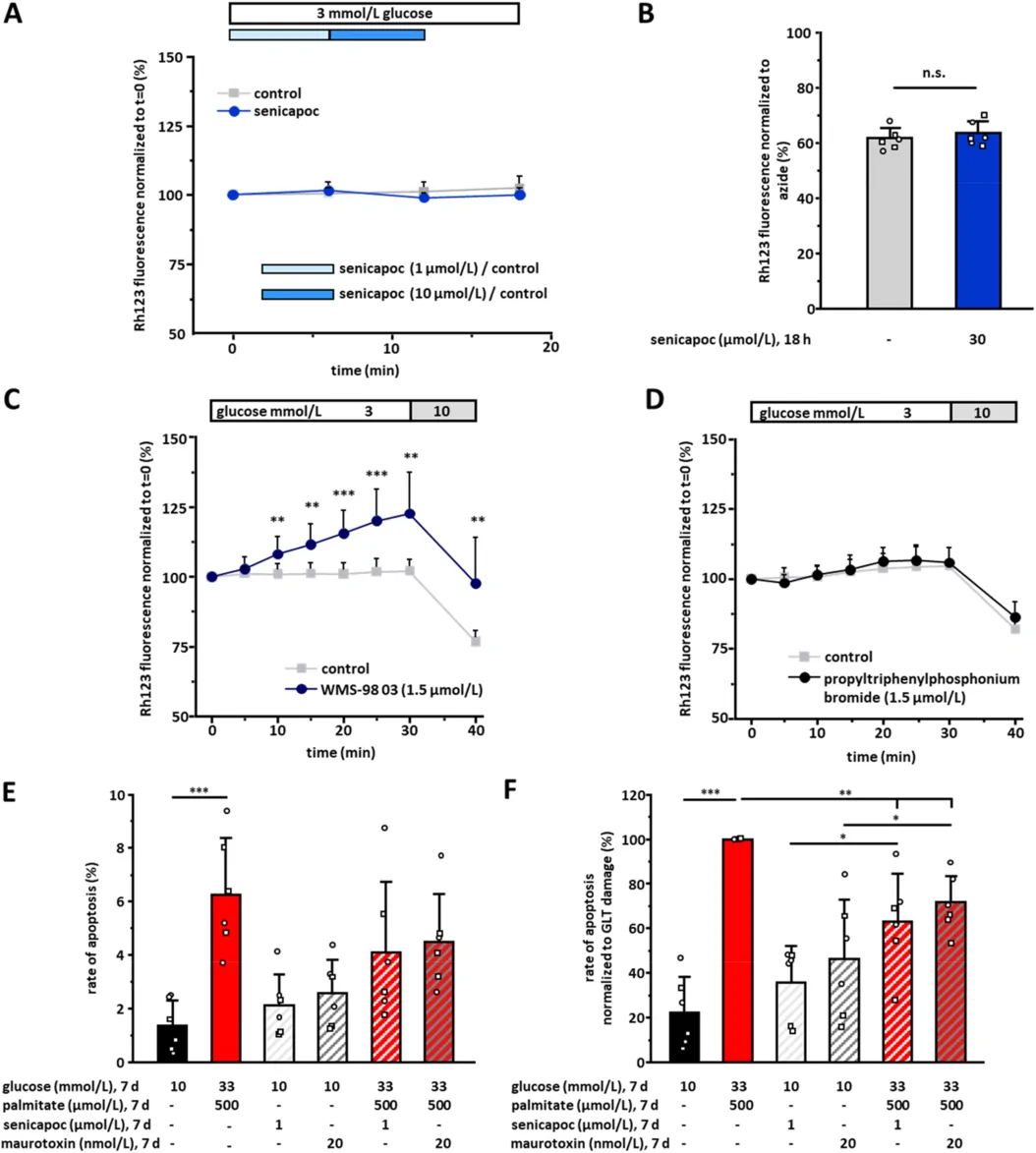
(A–D) Mitochondrial membrane potential measurements in beta cells. (A) Senicapoc (1 µmol/L) was added at *t* = 0 min for 6 min, followed by 6 min of senicapoc (10 µmol/L). In the controls only the solvent DMSO (1:1000) was added. Senicapoc had no acute effect on the mitochondrial membrane potential. Data were normalized to fluorescence intensity at *t* = 0 min, and the curves represent the mean data from eight independent experiments per condition (male/female: 3/5 preparations). (B) Senicapoc (30 µmol/L) had no effect on the mitochondrial membrane potential after 18 h of preincubation. Data were normalized to the maximum of depolarization by 10 mmol/L sodium azide. (C/D) WMS‐98 03 (1.5 µmol/L), propyltriphenylphosphonium bromide (1.5 µmol/L), or the solvent DMSO (1:1000) was added at *t* = 0 min for 30 min. Thereafter, glucose concentration was elevated from 3 to 10 mmol/L in the continued presence of the test compounds. WMS‐98 03 depolarized the mitochondrial membrane potential, whereas propyltriphenylphosphonium had no effect. Data were normalized to fluorescence intensity at *t* = 0 min. The curves represent the mean data from 10 (C, male/female: 6/4) and 5 (D, male/female: 3/2) independent preparations per condition. **p < 0.01, ***p < 0.001 versus control. (E/F) Islet cells were incubated for 7 days with the indicated conditions. Apoptotic cells were marked by TUNEL and nuclei by bisbenzimide staining. Glucolipotoxicity elevated the rate of apoptosis, while senicapoc (1 µmol/L) and maurotoxin (20 nmol/L) tended to protect from this damage. The rate of apoptosis is presented as percentage of all bisbenzimide‐positive cells in (E) and normalized to the effect of glucolipotoxicity in (F). Squares represent data points acquired from male mice, circles show data points from female mice (male/female: 3/3). *p < 0.05, **p < 0.01, ***p < 0.001 as indicated.

## 3 Discussion

This study demonstrates a shift K_Ca_3.1 channels undergo regarding the dependency of the Ca^2+^ source for channel activation and the significance for beta cell function and survival in response to metabolic stress.

### 3.1 Interplay Between K_Ca_3.1 Current and Ca^2+^ Influx

The activation of K_Ca_3.1 channels is due to a rise in [Ca^2+^]_i_ [23, 24], which can be controlled by several mechanisms such as Ca^2+^ influx via ion channels, ER Ca^2+^ storage, mitochondrial Ca^2+^ uptake, and Ca^2+^ efflux via Ca^2+^ pumps [25, 26, 27]. In pancreatic beta cells, the elevation in [Ca^2+^]_i_ for K_Ca_ channel activation is mainly driven by Ca^2+^ influx via Ca_V_ channels, predominantly l‐type Ca_v_ channels, and Ca^2+^ release from the ER, but NMDARs can also play a role [13, 14, 16]. We now show that the relevance of l‐type Ca_v_ channel‐dependent Ca^2+^ influx for K_Ca_3.1 channel activation is high in beta cells cultured under “physiological” conditions but becomes distinctly less important for metabolically stressed cells. Interestingly, Ca^2+^ flux through GluN2B‐containing NMDAR appears to be the main factor for K_Ca_3.1 channel activation in metabolically stressed cells. A possible explanation for this change in ion channel coupling could be shifts in microdomain composition. K_Ca_3.1 channels are known to have a short half‐life of 60–90 min before being internalized and degraded in HEK cells, and HMEC‐1 cells and trafficking of K_Ca_3.1 channels to the cell membrane in epithelial cells is intensively investigated, revealing several structures like the multi‐protein exocyst complex, the cytoskeleton, or peptide Lv, that are important for the assembly and trafficking to the cell membrane [28, 29, 30, 31]. Taking these complex mechanisms into consideration, it becomes plausible that disruption of cell metabolism might result in changes in ion channel microdomain composition, which lead to altered coupling of K_Ca_3.1 to Ca^2+^‐conducting ion channels. Furthermore, it was shown that pancreatic beta cells exhibit Ca^2+^ microdomains and Ca_V_ channel clusters colocalized with insulin vesicles [32, 33, 34, 35]. Taken together, this enables the hypothesis that K_Ca_3.1 channels, which are highly dependent on l‐type Ca_V_‐dependent Ca^2+^ flux, are physiologically placed near these Ca_V_ channel clusters and potentially lose this close proximity due to metabolic stress, resulting in altered ion channel coupling and a potentially shifted function. Further studies are necessary to elucidate if direct interactions between the aforementioned ion channels are involved.

Importantly, we did not observe a significant decrease in K_slow_ current due to glucolipotoxicity but a significant reduction in the contribution of K_Ca_3.1 current to K_slow_ occurring despite of the channel upregulation previously reported [16]. These data lead to the hypothesis that metabolic stress induces upregulation of K_Ca_3.1 channels while simultaneously impairing their function within the islet cell network (see Figure 6 for a summary of our findings and the resulting hypothesis). Such a conclusion is further supported by the inability of K_Ca_3.1 channel inhibition to further elevate electrical activity in whole islets that are in a phase of hyperexcitability in response to excessive supply of glucose and lipids representing early phases in the development of type 2 diabetes mellitus.

**Figure 6:**
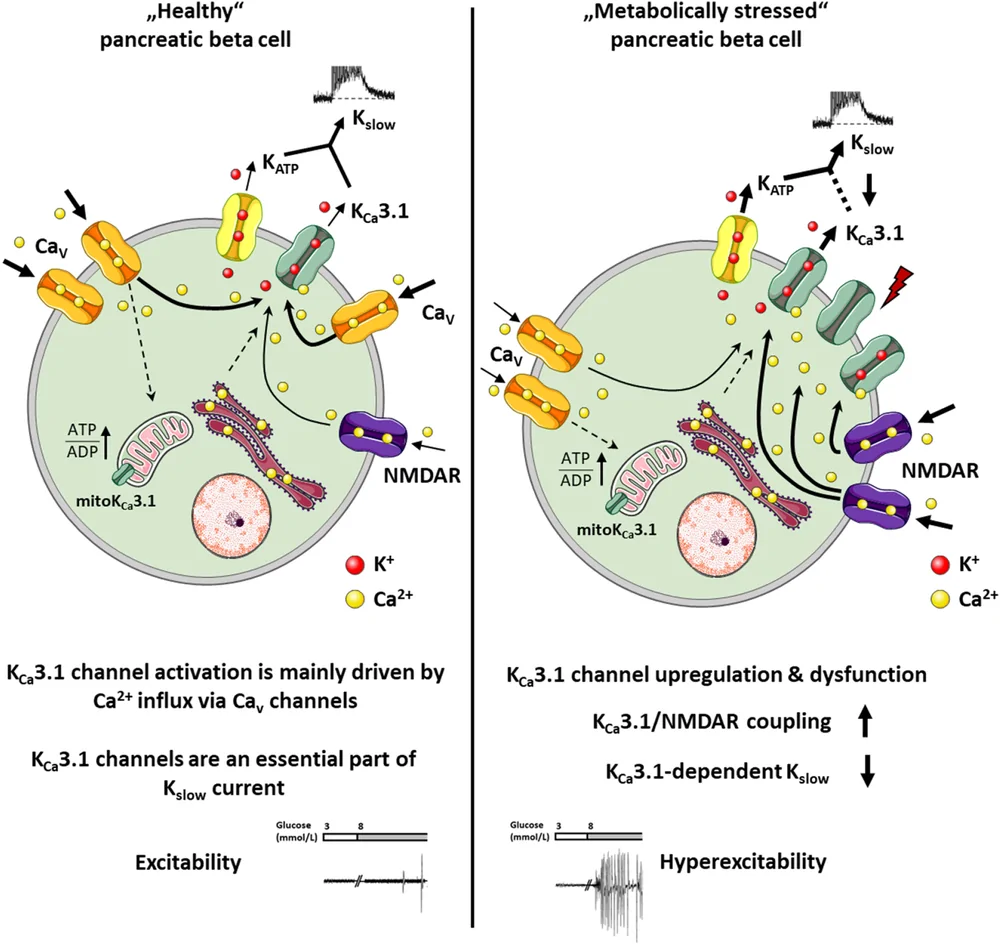
Scheme illustrating the changes regarding K_Ca_3.1 channel function and interaction in response to metabolic stress.

### 3.2 K_Ca_3.1 Channels in the Context of Larger Networks for Programmed Cell Death

Glucotoxicity and lipotoxicity activate several pathways of programmed cell death such as disruption of ER Ca^2+^ handling leading to ER stress, excessive mitochondrial ROS generation or inflammation in pancreatic beta cells [36, 37, 38]. Furthermore, apoptotic signalling is accompanied by other events like cell volume decrease, partly triggered by K^+^ efflux [39]. Increased K^+^ efflux via K^+^ channels and concomitant reduction of intracellular K^+^ is known to set aside the inhibitory influence of K^+^ on caspases. We previously showed that blocking K_Ca_3.1 channels by senicapoc partly protects islet cells from glucolipotoxicity‐induced apoptosis [16]. K_Ca_3.1 channels can be involved in a variety of pro‐apoptotic mechanisms. K^+^ efflux is a known activator of the NLRP3 inflammasome, and it was recently shown that K_Ca_3.1 channels influence NLRP3 inflammasome activity in macrophages [40, 41]. NLRP3 is functionally expressed in pancreatic beta cells and considered to take part in IL‐1β secretion resulting in pyroptosis, even though it is also reported that NLRP3 activity is not involved in beta cell death [38, 42, 43, 44]. Nevertheless, it appears tempting to hypothesize that an increased and dysregulated K^+^ efflux through K_Ca_3.1 channels elicited by glucolipotoxicity connects the reported interplay between K_Ca_3.1 channel activity and inflammation with cell death by activation of such a pathway [15]. Glucolipotoxicity induces ER stress, which is a main factor for beta cell death. ER stress in beta cells is evoked by accumulation of misfolded proteins mainly due to the high insulin demand. Recently, it was shown that inhibition of K_Ca_3.1 channels reduces ER stress in alveolar epithelial cells of sheep in a model for pulmonary fibrosis, and similar results were acquired for astrocytes [45, 46]. This offers a potential relevance of ER stress amelioration as a mechanism of apoptosis reduction by K_Ca_3.1 inhibition in beta cells. Furthermore, ion concentrations are a main factor for the regulation of cell volume. Cells undergoing apoptosis show a characteristic shrinkage called apoptotic volume decrease in which ion fluxes are strongly involved [39]. Several ion channels are considered to take part in this process, and for T lymphocytes it was shown that inhibition of K_Ca_3.1 is preventing apoptotic volume decrease [47], thereby offering a third possible proapoptotic property of dysregulated K_Ca_3.1 channels in metabolically stressed pancreatic beta cells. The NMDAR and its various subunits are attracting growing interest as a factor for the dysfunction of pancreatic beta cells [16, 20, 48]. Interactions between NMDARs and K_Ca_3.1 channels seem to depend on the composition of the NMDAR [16]. Interestingly, our new data prove that Ca^2+^ entering the cell through GluN2B‐containing NMDARs becomes more relevant for the activation of K_Ca_3.1 current in glucolipotoxicity‐challenged beta cells without coupling to the—in terms of Ca^2+^ overload—“protective” hyperpolarizing K_slow_ current. It is known that overactivation of NMDARs induces apoptosis and that glucolipotoxicity‐induced apoptosis is partly mediated by GluN2B‐containing NMDARs. Protection against cell death in response to metabolic stress was reported for two structurally unrelated NMDAR inhibitors targeting GluN2B [20]. Recently, we showed that GluN2B mediates ER stress and ROS generation via mitochondria and NADPH oxidase activity. Now we are extending this observation by the changed function and coupling of K_Ca_3.1 channels to GluN2B‐containing NMDARs due to glucolipotoxicity, making it a conceivable downstream mediator of apoptotic signalling pathways in response to strong or prolonged activation of the NMDAR.

### 3.3 Plasma Membrane and Mitochondrial K_Ca_3.1 Channels

Regarding cell death in the context of cancer, mitochondrial K_Ca_ channels have been under consideration for some time now, including pancreatic ductal adenocarcinoma [22, 49]. For the endocrine pancreas, it is not known whether mitoK_Ca_3.1 channels play a regulatory role. Preliminary data from our group indicate a weak expression of mitoK_Ca_3.1 channels in the insulin‐secreting cell line MIN6 (not shown), and we previously reported a co‐staining of mitochondria and K_Ca_3.1 channels by a channel‐targeted fluorescent dye in mouse islet cells [11]. As senicapoc might block not only plasma membrane but also mitochondrial K_Ca_3.1 channels in islet cells, we now tested whether the mitochondrial membrane potential is altered by senicapoc. Our data show that this is not the case even with a high senicapoc concentration. Considered together with the anti‐apoptotic effect observed with the membrane‐impermeant maurotoxin, the reduction of stress‐mediated islet cell apoptosis is ascribed to inhibition of the plasma membrane K_Ca_3.1 channel. Nevertheless, our current investigation for the first time provides good evidence for a functional role of mitoK_Ca_3.1 channels in pancreatic beta cells. With the modified, mitochondria‐targeted senicapoc, where a driving force for mitochondrial accumulation is provided by a positively charged and lipophilic triphenylphosphonium group, we observed a mitochondrial depolarization that develops within 10–20 min after addition of the compound to the bath solution. One limitation of this study is that experiments were performed with mouse and not with human cells. However, our observation nicely fits with data from a human melanoma cell line, for which rapid mitochondrial depolarization was described in a similar approach with the structurally related K_Ca_3.1 inhibitor TRAM‐34 [22]. The importance of the channels for the endocrine pancreas remains to be elucidated and is a topic of our ongoing research.

## 4 Conclusion

The relevance of the K_Ca_3.1 channel for the regulation of electrical activity of beta cells and islets is diminished, while the channel becomes a mediator of glucolipotoxicity‐induced apoptosis. The negative impact on cell mass is sensitive to inhibitors of the plasma membrane‐located K_Ca_3.1 channel and seems to involve an altered coupling to Ca^2+^‐conducting ion channels.

## 5 Experimental

### 5.1 Animals and Cell Culture

Pancreatic islets or islet cells from adult male and female C57BL/6 N wildtype or SUR1^–/–^ mice (own breeding and Charles River, Germany) were used for experiments. The data of male and female mice were pooled, as there were no obvious sex‐dependent differences (see Supporting Information S1: Figure 5 for I/V curves and Supporting Table for comparative analysis of other experiments). The principles of laboratory animal care were followed according to German laws (Az. 53.5.32.71/MS‐12668, health and veterinary office Münster, Germany). Mice were killed with CO_2_ and islets were isolated by collagenase digestion. Single cells were obtained by trypsin‐EDTA (Thermo Fisher, Germany) digestion of islets. Islets or cells were cultured in RPMI 1640 (Thermo Fisher, Germany) medium (11.1 mmol/L glucose) supplemented with 10% fetal calf serum (FCS, Fisher Scientific, Germany), 100 U/mL penicillin and 100 µg/mL streptomycin (Thermo Fisher, Germany). For glucolipotoxic conditions, 100 µmol/L palmitate, bound to serum albumin (0.056%), was added to the standard culture medium, and the glucose concentration was adjusted to 25 mmol/L. For apoptosis experiments, glucolipotoxic conditions were modified to 500 µmol/L BSA‐bound palmitate and 33 mmol/L glucose. Stress conditions were applied for 7 days before determination of apoptosis, up to 13 days for MEA recordings, and for 48 h in all other experiments.

### 5.2 Solutions and Chemicals

External solutions for whole cell recordings (in mmol/L): 138 NaCl, 5.6 KCl, 2.6 CaCl_2_, 1.2 MgCl_2_, 5 HEPES, 5 glucose, pH 7.4 adjusted with NaOH. External solution for K_slow_ current recordings (in mmol/L): 140 NaCl, 3.6 KCl, 2.5 CaCl_2_, 2 NaHCO_3_, 0.5 NaH_2_PO_4_, 0.5 MgSO_4_, 5 HEPES, 15 glucose, pH 7.4 adjusted with NaOH. Pipette solution for whole‐cell recordings (in mmol/L): 140 KCl, 1 MgCl_2_, 1 CaCl_2_, 10 EGTA, 3 Na_2_‐ATP, 10 HEPES, pH 7.2 adjusted with KOH. Pipette solution for K_slow_ current recordings (in mmol/L): 10 NaCl, 10 KCl, 70 K_2_SO_4_, 4 MgCl_2_, 2 CaCl_2_, 10 EGTA, 5 HEPES. Amphotericin B was added right before the experiment (0.5 mg/L). Solution for MEA measurements and monitoring of mitochondrial membrane potential: 140 NaCl, 5 KCl, 1.2 MgCl_2_, 2.5 CaCl_2_, 10 HEPES, glucose as indicated. Ro 25‐6981 and nifedipine were obtained from Merck, Germany.

### 5.3 Synthesis

#### 5.3.1 General

All chemicals were purchased in the highest available quality and used without further purification. Temperature was controlled with a magnetic stirrer MR 3001 K (Heidolph, Schwalbach, Germany) or RCT CL (IKA, Staufen, Germany), together with a temperature controller EKT HeiCon (Heidolph) or VT‐5 (VWR) and a PEG or silicone bath. All solvents were of analytical or technical grade quality. Demineralized water was used. CH_2_Cl_2_ was distilled from CaH_2_. Thin‐layer chromatography (TLC): tlc silica gel 60 F254 on aluminum sheets (VWR). Automated flash chromatography: IsoleraTM Spektra One (Biotage); parentheses include: cartridge size, eluent, fractions size was always 20 mL. Melting point: Melting point system MP50 (Mettler Toledo, Gießen, Germany), open capillary, uncorrected. MS: MicroTOFQII mass spectrometer (Bruker Daltonics, Bremen, Germany); deviations of the found exact masses from the calculated exact masses were 10 ppm or less; the data were analyzed with DataAnalysis (Bruker Daltonics). NMR: NMR spectra were recorded in deuterated solvents on Agilent DD2 400 MHz and 600 MHz spectrometers (Agilent, Santa Clara, CA, USA); chemical shifts (δ) are reported in parts per million (ppm) against the reference substance tetramethylsilane and calculated using the solvent residual peak of the undeuterated solvent; coupling constants are given with 0.5 Hz resolution; assignment of ^1^H and ^13^C NMR signals was supported by 2‐D NMR techniques where necessary. IR: FT/IR IR Affinity®−1 spectrometer (Shimadzu, Düsseldorf, Germany) using ATR technique.

#### 5.3.2 HPLC Method to Determine the Purity

DIONEX UltiMate 3000; UV detector: VWD‐3400RS; autosampler: ACC‐3000T; pump: LPG‐3400SD; degasser: DG‐1210; Method: column: LiChrospher 60 RP‐select B (5 µm), 250 × 4 mm; flow rate: 1.00 mL/min; injection volume: 5.0 µL; detection at *λ* = 210 nm; solvents: A: water with 0.05% (v/v) trifluoroacetic acid; B: acetonitrile with 0.05% (v/v) trifluoroacetic acid: gradient elution: (A%): 0–4 min: 90%, 4–29 min: gradient from 90% to 0%, 29–31 min: 0%, 31–31.5 min: gradient from 0% to 90%, 31.5–40 min: 90%. See Supporting Information S1: Figure 4A for chromatogram.

#### 5.3.3 Synthetic Procedures

**Synthesis of (4‐{4‐[1‐Carbamoyl‐1,1‐bis(4‐fluorophenyl)methyl]phenoxy}−4‐oxobutyl) triphenylphosphoniumbromide (WMS‐98 03)**


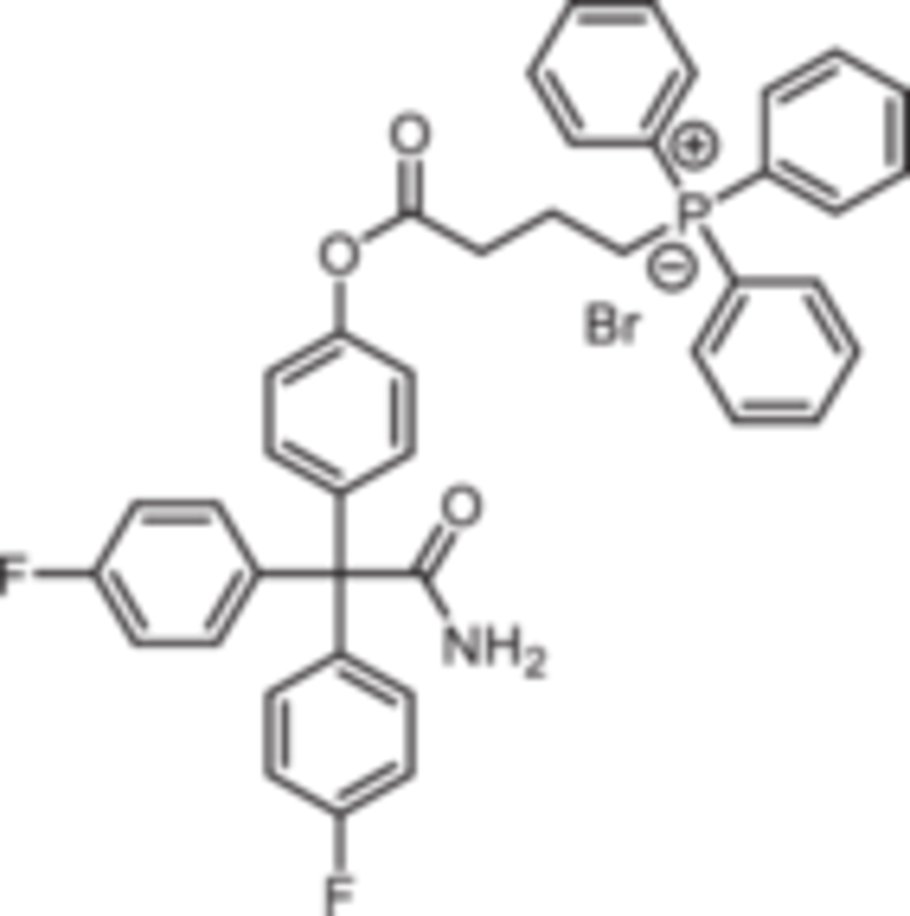


(3‐Carboxypropyl)triphenylposphonium bromide (73 mg, 0.17 mmol, 1.1 eq.) and DMAP (4 mg, 0.03 mmol, 0.2 eq.) were dissolved in dry CH_2_Cl_2_ (5 mL). EDC∙HCl (58 mg, 0.3 mmol, 2.0 eq.) was added, and the mixture was stirred at room temperature for 30 min. 2,2‐Bis(4‐fluorophenyl)−2‐(4‐hydroxyphenyl)acetamide (50 mg, 0.15 mmol, 1.0 eq.) was added, and the mixture was stirred over night at room temperature. The solvent was removed in vacuo, and the resulting crude product was purified by automatic flash column chromatography (SiO_2_, 10 g, Ø=2.5 cm, gradient CH_2_Cl/CH_3_OH 100:0 → 90:10). Colorless solid, mp 143°C. yield 81.9 mg (73%). **TLC** *R*_f_ = 0.27 (CH_2_Cl/CH_3_OH 90:10). **Exact mass** (ESI): (*m/z*) = 670.2349 (calcd. 670.2317 for C_42_H_35_F_2_NO_3_P [M]^+^). ^**1**^ **H NMR** (600 MHz, DMSO‐*d*_6_): *δ* (ppm) = 1.82–1.94 (m, 2H, COCH_2_C*H*_2_), 2.84 (t, *J* = 6.8 Hz, 2H, COC*H*_2_), 3.63–3.72 (m, 2H, C*H*_2_P), 6.82 (s, 1H, N*H*_2_), 7.09 (d, *J* = 8.9 Hz, 2H, 3‐*H*, 5‐*H*_(OPh)_), 7.12–7.19 (m, 4H, 3‐*H*, 5‐*H*_(FPh)_), 7.19–7.25 (m, 6H, 2‐*H*, 6‐*H*_(FPh)_, 2‐*H*, 6‐*H*_(OPh)_), 7.60 (s, 1H, N*H*_2_), 7.75–7.81 (m, 6H, 2‐*H*, 6‐*H*_(PPh3)_), 7.81–7.88 (m, 6H, 3‐*H*, 5‐*H*_(PPh3)_), 7.88–7.95 (m, 3H, 4‐*H*_(PPh3)_). ^1**3**^ **C NMR** (151 MHz, DMSO‐*d*_6_): *δ* (ppm) = 17.6 (d, *J* = 3.0 Hz, COCH_2_ *C*H_2_), 19.8 (d, *J* = 51.9 Hz, 1 C, *C*H_2_P), 33.5 (d, *J* = 18.5 Hz, 1 C, *C*OCH_2_), 65.6 (1 C, *C*CONH_2_), 114.5 (d, *J* = 21.1 Hz, 4 C, C‐3, C‐5 _(FPh)_), 118.3 (d, *J* = 86.0 Hz, 3 C, C‐1 _(PPh3)_), 121.0 (2 C, C‐3, C‐5 _(OPh)_), 130.3 (d, *J* = 12.6 Hz, 6 C, C‐2, C‐6 _(PPh3)_), 131.0 (2 C, C‐2, C‐6 _(OPh)_), 132.0 (d, *J* = 8.1 Hz, 4 C, C‐2, C‐6 _(FPh)_), 133.6 (d, *J* = 10.0 Hz, 6 C, C‐3, C‐5 _(PPh3)_), 135.0 (d, *J* = 3.2 Hz, 3 C, C‐4 _(PPh3)_), 139.8 (d, *J* = 3.3 Hz, 2 C, C‐1 _(FPh3)_), 141.3 (1 C, C‐1 _(OPh)_), 148.7 (1 C, C‐4 _(OPh)_), 160.7 (d, *J* = 244.0 Hz, 2 C, C‐4 _(FPh)_), 170.6 (1 C, O*C*(= O)CH_2_), 173.7 (1 C, *C*ONH_2_). **IR**: ṽ (cm^−1^) = 3476 (N–H), 3055 (C─H_arom._), 2870 (C─H_aliph._), 1748 (C═O_ester_), 1674 (C═O_amide_), 1504 (C═C_arom._). **Purity** (HPLC): 94.7%, t_R_ = 23.9 min. See Supporting Information S1: Figure 4 (B and C) for NMR spectra.

### 5.4 Apoptosis Assay

The K_Ca_3.1 channel blockers senicapoc (Cayman Chemicals, USA) or maurotoxin (Alomone labs, Israel) were present during the 7‐day culture period in the indicated media. Islet cells were fixated with 3% PFA solution (Merck, Germany) dissolved in 100 mM HEPES buffer and permeabilized with 0.1% Triton‐X (Carl Roth, Germany) solution dissolved in 0.1% sodium citrate solution. Apoptotic cells were determined using a TUNEL assay (in situ cell death detection kit, fluorescein, Roche Diagnostics, Germany). Cells were stained according to the manufacturer's protocol, and apoptotic cells were identified by fluorescein fluorescence excited at 480 nm excitation wavelength. The total number of cells was detected by nuclear staining with bisbenzimide (Hoechst 33258, Merck, Germany) at 380 nm excitation wavelength. Each condition was prepared in duplicate per mouse islet isolation. A minimum of 130 islet cells in 10 randomly selected areas of each culture dish was counted.

### 5.5 Electrophysiology

Channel blockers were applied acutely after culture in the indicated media. Single islet cells or small cell clusters were used for patch‐clamp recordings. Borosilicate glass capillaries were used for pulling pipettes (3‐5 MΩ). Currents were recorded with an EPC‐10 patch‐clamp amplifier (HEKA, Germany) and analyzed with Fitmaster (HEKA, Germany). Outward K^+^ currents were measured in the whole‐cell mode with increasing voltage steps (10 mV, 500 ms per step) from −60 mV to 80 mV starting from a holding potential of −70 mV. The mean outward current was calculated as the current from 7.5 ms to 492.5 ms of each voltage step. Beta cells were identified by the absence of A‐current. K_slow_ currents were recorded in the perforated‐patch configuration by applying a train of 26 voltage ramps starting from the holding potential of −40 mV rising to 0 mV and descending back to −40 mV within 200 ms for each ramp. After the train of voltage ramps, the cell was clamped at −40 mV for 20 s. Whole islet electrical activity was recorded with microelectrode arrays (MEA2100‐system with 60MEA200/30iR‐Ti‐gr, Multi Channel Systems, Germany). Islets were kept in external solution (glucose 3 mmol/L) for up to 1 h before putting them individually on an electrode with a holding pipette (441030CH, Reproline, Germany). The glucose concentration was elevated to 6 or 8 mmol/L when islets showed no electrical activity at 3 mmol/L glucose concentration. For data acquisition, a low‐pass filter at 20 Hz and a sample rate of 1000 Hz were used (Beta‐Screen Experimenter, Multi Channel Systems). The fraction of plateau phase (FOPP) was analyzed as the relative time of electrical activity. With 6 or 8 mmol/L glucose and after addition of senicapoc the total 10 min of data acquisition were analyzed.

### 5.6 Mitochondrial Membrane Potential

Channel blockers were applied acutely or for an 18‐h period as indicated in the respective figures. The mitochondrial membrane potential was measured by rhodamine 123 (Rh123; Merck, Germany). Islet cells were maintained in external solution containing 3 mmol/L glucose for 15 min prior to incubation with 52 µmol/L Rh123 for 15 min at 37°C in the dark. The Rh123 fluorescence intensity was detected at 488 nm excitation wavelength, and images were analyzed in 5 or 6 min intervals. Compounds were added for the indicated periods. In acute measurements, mitochondrial membrane hyperpolarization was induced at the end of each experiment by increasing the glucose concentration from 3 to 10 mmol/L, serving as an indicator of intact cellular metabolism. Maximal mitochondrial depolarization was induced by 10 mmol/L sodium azide (Merck, Germany) at the end of each experiment. If a cell was not responsive to sodium azide, it was discarded. Each condition was evaluated at least in duplicate (two independent culture dishes) per mouse islet isolation.

### 5.7 Statistics

All data were acquired from islets or islet cells from at least three independent mouse preparations per experimental setup and are depicted as mean ± SD. All data sets were tested for normal distribution. Normally distributed data were analyzed by Student's *t* test or One‐way ANOVA with a Student–Newman–Keuls *post hoc* test. p‐values lower than 0.05 were considered as statistically significant. In case normal distribution could not be assumed, a non‐parametric test was performed to verify obtained statistical significances. When two experimental groups were compared, a Wilcoxon–Mann–Whitney test was used (Figure 3C). In case more than two groups were analysed, a Kruskal‐Wallis test followed by Dunn´s test was carried out.

## Conflicts of Interest

The authors declare no conflicts of interest.

## Supporting information


Supporting File 1

Supporting File 2

## Data Availability

The data that support the findings of this study are available from the corresponding author upon reasonable request.

## References

[1] P. A. Smith , K. Bokvist , P. Arkhammar , P. O. Berggren , and P. Rorsman , “Delayed Rectifying and Calcium‐Activated K^+^ Channels and Their Significance for Action Potential Repolarization in Mouse Pancreatic Beta‐Cells,” Journal of General Physiology 95, no. 6 (1990): 1041–1059, 10.1085/jgp.95.6.1041. PMC2216351 2197368

[2] D. A. Jacobson , A. Kuznetsov , J. P. Lopez , S. Kash , C. E. Ämmälä , and L. H. Philipson , “Kv2.1 Ablation Alters Glucose‐Induced Islet Electrical Activity, Enhancing Insulin Secretion,” Cell Metabolism 6, no. 3 (2007): 229–235, 10.1016/j.cmet.2007.07.010. PMC2699758 17767909

[3] S.‐N. Yang , Y. Shi , G. Yang , Y. Li , J. Yu , and P.‐O. Berggren , “Ionic Mechanisms in Pancreatic β Cell Signaling,” Cellular and Molecular Life Sciences 71, no. 21 (2014): 4149–4177, 10.1007/s00018-014-1680-6. PMC11113777 25052376

[4] G. Drews , P. Krippeit‐Drews , and M. Düfer , Islets of Langerhans, eds. M. S. Islam (Springer Netherlands, 2015, chapter Electrophysiology of Islet Cells).

[5] M. Düfer , B. Gier , D. Wolpers , P. Krippeit‐Drews , P. Ruth , and G. Drews , “Enhanced Glucose Tolerance by SK4 Channel Inhibition in Pancreatic Β‐Cells,” Diabetes 58, no. 8 (2009): 1835–1843, 10.2337/db08-1324. PMC2712794 19401418

[6] N. A. Tamarina , Y. Wang , L. Mariotto , et al., “Small‐Conductance Calcium‐Activated K^+^ Channels Are Expressed in Pancreatic Islets and Regulate Glucose Responses,” Diabetes 52, no. 8 (2003): 2000–2006, 10.2337/diabetes.52.8.2000. 12882916

[7] B. Ribalet , G. T. Eddlestone , and S. Ciani , “Metabolic Regulation of the K(ATP) and a Maxi‐K(V) Channel in the Insulin‐Secreting RINm5F Cell,” Journal of General Physiology 92, no. 2 (1988): 219–237, 10.1085/jgp.92.2.219. PMC2228897 3049931

[8] B. King , A. P. Rizwan , H. Asmara , et al., “IKCa Channels Are a Critical Determinant of the Slow AHP in CA1 Pyramidal Neurons,” Cell Reports 11, no. 2 (2015): 175–182, 10.1016/j.celrep.2015.03.026. 25865881

[9] A.‐L. Hasenau , G. Nielsen , C. Morisseau , B. D. Hammock , H. Wulff , and R. Köhler , “Improvement of Endothelium‐Dependent Vasodilations by SKA‐31 and SKA‐20, Activators of Small‐ and Intermediate‐Conductance Ca^2^ ^+^−Activated K^+^−Channels,” Acta Physiologica 203, no. 1 (2011): 117–126, 10.1111/j.1748-1716.2010.02240.x. PMC3139805 21362152

[10] H. Wulff and R. Köhler , “Endothelial Small‐Conductance and Intermediate‐Conductance K_Ca_ Channels: An Update on Their Pharmacology and Usefulness as Cardiovascular Targets,” Journal of Cardiovascular Pharmacology 61, no. 2 (2013): 102–112, 10.1097/FJC.0b013e318279ba20. PMC3565027 23107876

[11] B. Soret , J. Hense , S. Lüdtke , I. Thale , A. Schwab , and M. Düfer , “Pancreatic K_Ca_3.1 Channels in Health and Disease,” Biological Chemistry 404, no. 4 (2023): 339–353, 10.1515/hsz-2022-0232. 36571487

[12] J. Maylie , C. T. Bond , P. S. Herson , W.‐S. Lee , and J. P. Adelman , “Small Conductance Ca^2+^‐Activated K^+^−Channels and Calmodulin,” Journal of Physiology 554, no. Pt 2 (2004): 255–261, 10.1113/jphysiol.2003.049072. PMC1664776 14500775

[13] S. O. Göpel , T. Kanno , S. Barg , et al., “Activation of Ca^2+^−Dependent K^+^ Channels Contributes to Rhythmic Firing of Action Potentials in Mouse Pancreatic β Cells,” Journal of General Physiology 114, no. 6 (1999): 759–770, 10.1085/jgp.114.6.759. PMC2230648 10578013

[14] P. B. Goforth , R. Bertram , F. A. Khan , M. Zhang , A. Sherman , and L. S. Satin , “Calcium‐Activated K^+^ Channels of Mouse β‐cells Are Controlled by Both Store and Cytoplasmic Ca^2+^ ,” Journal of General Physiology 120, no. 3 (2002): 307–322, 10.1085/jgp.20028581. PMC2229522 12198088

[15] Z.‐D. Pang , Y. Wang , X.‐J. Wang , et al., “KCa3.1 Channel Mediates Inflammatory Signaling of Pancreatic β Cells and Progression of Type 2 Diabetes Mellitus,” FASEB Journal 33, no. 12 (2019): 14760–14771, 10.1096/fj.201901329RR. 31690106

[16] H. Noguera Hurtado , J. Hense , N. Klöpper , J. Osthues , B. Wünsch , and M. Düfer , “Targeting GluN2B‐containing NMDA Receptors to Interact With Stress Pathways and Ca^2+^‐Regulated K^+^ Channels in Murine Islets of Langerhans,” European Journal of Pharmacology 1007 (2025): 178223, 10.1016/j.ejphar.2025.178223. 41075917

[17] M. T. Dickerson , A. M. Bogart , M. K. Altman , et al., “Cytokine‐Mediated Changes in K^+^ Channel Activity Promotes An Adaptive Ca^2+^ Response That Sustains β‐cell Insulin Secretion During Inflammation,” Scientific Reports 8, no. 1 (2018): 1158, 10.1038/s41598-018-19600-x. PMC5773563 29348619

[18] B. S. Jensen , D. Strobaek , P. Christophersen , et al., “Characterization of the Cloned Human Intermediate‐Conductance Ca2^+^−activated K^+^ Channel,” American Journal of Physiology 275, no. 3 (1998): 848–856, 10.1152/ajpcell.1998.275.3.C848. 9730970

[19] X.‐T. Li , X.‐Q. Li , X.‐M. Hu , and X.‐Y. Qiu , “The Inhibitory Effects of Ca^2+^ Channel Blocker Nifedipine on Rat Kv2.1 Potassium Channels,” PLoS One 10, no. 4 (2015): e0124602, 10.1371/journal.pone.0124602. PMC4404097 25893973

[20] A. Gresch , H. Noguera Hurtado , L. Wörmeyer , et al., “Selective Inhibition of N‐Methyl‐D‐Aspartate Receptors With GluN2B Subunit Protects β Cells Against Stress‐Induced Apoptotic Cell Death,” Journal of Pharmacology and Experimental Therapeutics 379, no. 3 (2021): 235–244, 10.1124/jpet.121.000807. 34593560

[21] X.‐T. Huang , C. Li , X.‐P. Peng , et al., “An Excessive Increase in Glutamate Contributes to Glucose‐Toxicity in β‐cells via Activation of Pancreatic NMDA Receptors in Rodent Diabetes,” Scientific Reports 7 (2017): 44120, 10.1038/srep44120. PMC5356012 28303894

[22] M. Bachmann , A. Rossa , T. Varanita , et al., “Pharmacological Targeting of the Mitochondrial Calcium‐Dependent Potassium Channel K_Ca_3.1 Triggers Cell Death and Reduces Tumor Growth and Metastasis In Vivo,” Cell Death & Disease 13, no. 12 (2022): 1055, 10.1038/s41419-022-05463-8. PMC9768205 36539400

[23] L. Sforna , A. Megaro , M. Pessia , F. Franciolini , and L. Catacuzzeno , “Structure, Gating and Basic Functions of the Ca^2+^‐Activated K Channel of Intermediate Conductance,” Current Neuropharmacology 16, no. 5 (2018): 608–617, 10.2174/1570159X15666170830122402. PMC5997868 28875832

[24] B. Fioretti , E. Castigli , M. R. Micheli , et al., “Expression and Modulation of the Intermediate‐ Conductance Ca^2+^−Activated K^+^ Channel in Glioblastoma GL‐15 Cells,” Cellular Physiology and Biochemistry 18, no. 1–3 (2006): 47–56, 10.1159/000095135. 16914889

[25] I. X. Zhang , M. Raghavan , and L. S. Satin , “The Endoplasmic Reticulum and Calcium Homeostasis in Pancreatic Beta Cells,” Endocrinology 161, no. 2 (2020): 1–14, 10.1210/endocr/bqz028. PMC7028010 31796960

[26] P. V. Sabatini , T. Speckmann , and F. C. Lynn , “Friend and Foe: β‐cell Ca^2+^ Signaling and the Development of Diabetes,” Molecular Metabolism 21 (2019): 1–12, 10.1016/j.molmet.2018.12.007. PMC6407368 30630689

[27] P. Gilon , H.‐Y. Chae , G. A. Rutter , and M. A. Ravier , “Calcium Signaling in Pancreatic β‐cells in Health and in Type 2 Diabetes,” Cell Calcium 56, no. 5 (2014): 340–361, 10.1016/j.ceca.2014.09.001. 25239387

[28] Y. Gao , C. M. Balut , M. A. Bailey , G. Patino‐Lopez , S. Shaw , and D. C. Devor , “Recycling of the Ca^2+^‐Activated K^+^ Channel, K_Ca_2.3, Is Dependent Upon RME‐1, Rab35/EPI64C, and An N‐Terminal Domain,” Journal of Biological Chemistry 285, no. 23 (2010): 17938–17953, 10.1074/jbc.M109.086553. PMC2878556 20360009

[29] M. J. E. Logue , R. E. Farquhar , Y. Eckhoff‐Björngard , et al., “The Exocyst Complex Is Required for the Trafficking and Delivery of K_Ca_3.1 to the Basolateral Membrane of Polarized Epithelia,” American Journal of Physiology‐Cell Physiology 324, no. 6 (2023): C1249–C1262, 10.1152/ajpcell.00374.2022. PMC10243536 37125772

[30] R. E. Farquhar , E. Rodrigues , and K. L. Hamilton , “The Role of the Cytoskeleton and Myosin‐Vc in the Targeting of K_Ca_3.1 to the Basolateral Membrane of Polarized Epithelial Cells,” Frontiers in Physiology 7 (2016): 639. 10.3389/fphys.2016.00639. PMC5209343 28101059

[31] D. L. Pham , A. Niemi , R. Blank , et al., “Peptide Lv Promotes Trafficking and Membrane Insertion of K_Ca_3.1 Through the MEK1‐ERK and PI3K‐Akt Signaling Pathways,” Cells 12, no. 12 (2023): 1651, 10.3390/cells12121651. PMC10296961 37371121

[32] G. Langlhofer , A. Kogel , and M. Schaefer , “Glucose‐Induced [Ca^2+^]i Oscillations in β Cells Are Composed of Trains of Spikes Within a Subplasmalemmal Microdomain,” Cell Calcium 99 (2021): 102469, 10.1016/j.ceca.2021.102469. 34509871

[33] N. R. Gandasi , P. Yin , M. Riz , et al., “Ca^2+^ Channel Clustering With Insulin‐Containing Granules Is Disturbed in Type 2 Diabetes,” Journal of Clinical Investigation 127, no. 6 (2017): 2353–2364, 10.1172/JCI88491. PMC5451232 28481223

[34] G. A. Rutter , T. Tsuboi , and M. A. Ravier , “Ca^2+^ Microdomains and the Control of Insulin Secretion,” Cell Calcium 40, no. 5–6 (2006): 539–551, 10.1016/j.ceca.2006.08.015. 17030367

[35] F. Xia , X. Gao , E. Kwan , et al., “Disruption of Pancreatic β‐Cell Lipid Rafts Modifies Kv2.1 Channel Gating and Insulin Exocytosis,” Journal of Biological Chemistry 279, no. 23 (2004): 24685–24691, 10.1074/jbc.M314314200. 15073181

[36] M. Lytrivi , A.‐L. Castell , V. Poitout , and M. Cnop , “Recent Insights into Mechanisms of β‐Cell Lipo‐ and Glucolipotoxicity in Type 2 Diabetes,” Journal of Molecular Biology 432, no. 5 (2020): 1514–1534, 10.1016/j.jmb.2019.09.016. PMC7073302 31628942

[37] D. A. Cunha , P. Hekerman , L. Ladrière , et al., “Initiation and Execution of Lipotoxic ER Stress in Pancreatic Β‐Cells,” Journal of Cell Science 121, no. Pt 14 (2008): 2308–2318, 10.1242/jcs.026062. PMC3675788 18559892

[38] K. Maedler , P. Sergeev , F. Ris , et al., “Glucose‐Induced β Cell Production of IL‐1β Contributes to Glucotoxicity in Human Pancreatic Islets,” Journal of Clinical Investigation 110, no. 6 (2002): 851–860, 10.1172/JCI15318. PMC151125 12235117

[39] C. D. Bortner and J. A. Cidlowski , “Ions, the Movement of Water and the Apoptotic Volume Decrease,” Frontiers in Cell and Developmental Biology 8 (2020): 611211, 10.3389/fcell.2020.611211. PMC7723978 33324655

[40] V. Pétrilli , S. Papin , C. Dostert , A. Mayor , F. Martinon , and J. Tschopp , “Activation of the NALP3 Inflammasome Is Triggered by Low Intracellular Potassium Concentration,” Cell Death & Differentiation 14, no. 9 (2007): 1583–1589, 10.1038/sj.cdd.4402195. 17599094

[41] B. Zeng , Y. Huang , S. Chen , et al., “Dextran Sodium Sulfate Potentiates NLRP3 Inflammasome Activation by Modulating the K_Ca_3.1 Potassium Channel in a Mouse Model of Colitis,” Cellular & Molecular Immunology 19, no. 8 (2022): 925–943, 10.1038/s41423-022-00891-0. PMC9338299 35799057

[42] R. Zhou , A. Tardivel , B. Thorens , I. Choi , and J. Tschopp , “Thioredoxin‐Interacting Protein Links Oxidative Stress to Inflammasome Activation,” Nature Immunology 11, no. 2 (2010): 136–140, 10.1038/ni.1831. 20023662

[43] T. Liu , Q. Wang , Z. Du , et al., “The Trigger for Pancreatic Disease: NLRP3 Inflammasome,” Cell Death Discovery 9, no. 1 (2023): 246, 10.1038/s41420-023-01550-7. PMC10349060 37452057

[44] J. A. Wali , E. N. Gurzov , S. Fynch , et al., “Activation of the NLRP3 Inflammasome Complex Is Not Required for Stress‐Induced Death of Pancreatic Islets,” PLoS One 9, no. 11 (2014): e113128, 10.1371/journal.pone.0113128. PMC4236141 25405767

[45] U. E. Perera , L. Organ , S. Dewage , H. B. Derseh , A. Stent , and K. J. Snibson , “Increased Levels of ER Stress and Apoptosis in a Sheep Model for Pulmonary Fibrosis Are Alleviated by In Vivo Blockade of the K_Ca_3.1 Ion Channel,” Canadian Respiratory Journal 2021 (2021): 6683195, 10.1155/2021/6683195. PMC8004363 33828632

[46] Z. Yu , F. Dou , Y. Wang , L. Hou , and H. Chen , “Ca^2+^‐Dependent Endoplasmic Reticulum Stress Correlation With Astrogliosis Involves Upregulation of KCa3.1 and Inhibition of AKT/mTOR Signaling,” Journal of Neuroinflammation 15, no. 1 (2018): 316, 10.1186/s12974-018-1351-x. PMC6236981 30442153

[47] J. I. Elliott and C. F. Higgins , “IKCa1 Activity Is Required for Cell Shrinkage, Phosphatidylserine Translocation and Death in T Lymphocyte Apoptosis,” EMBO Reports 4, no. 2 (2003): 189–194, 10.1038/sj.embor.embor722. PMC1315824 12612610

[48] J. Marquard , S. Otter , A. Welters , et al., “Characterization of Pancreatic NMDA Receptors as Possible Drug Targets for Diabetes Treatment,” Nature Medicine 21, no. 4 (2015): 363–372, 10.1038/nm.3822. 25774850

[49] L. M. Todesca , M. Gerke , E. E. Bulk , et al., “Targeting K_Ca_3.1 Channels to Overcome Erlotinib Resistance in Non‐Small Cell Lung Cancer Cells,” Cell Death Discovery 10, no. 1 (2024): 2, 10.1038/s41420-023-01776-5. PMC10767088 38177097

