# Supplementary material for: Metabolic Stress Shifts the Function of KCa3.1 Channels in Pancreatic Beta Cells From a Regulator of Membrane Potential to a Proapoptotic Factor
Source: Arch Pharm (Weinheim). 2026 Oct 9;359(10):e70344. doi: 10.1002/ardp.70344 (PMC13651915; doi:10.1002/ardp.70344)
Supplement: Supplementary file 1 [file ARDP-359-e70344-s001.pdf]

ARCH PHARM
Archiv der Pharmazie

# Supplemental Material: Novel Compounds and Biological Screening Results

## Metabolic stress shifts the function of $K_{Ca}$3.1 channels in pancreatic beta cells from a regulator of membrane potential to a proapoptotic factor

Jurek Hense[1], Lena Beuke[1], Christina Kick[2], Bernhard Wünsch[2] and Martina Düfer[1]*

[1]University of Münster, Institute of Pharmaceutical and Medicinal Chemistry, Dept. of Pharmacology, Corrensstraße 48, 48149 Münster, Germany

[2]University of Münster, Institute of Pharmaceutical and Medicinal Chemistry, Corrensstraße 48, 48149 Münster, Germany

Correspondence: Martina Düfer, Dept. of Pharmacology, Institute of Pharmaceutical and Medicinal Chemistry, University of Münster, Corrensstraße 48, 48149 Münster, Germany, Phone: (+49) 251 83 33339, Email: martina.duefer@uni-muenster.de

| Compound No. | InChI | Biological Activity |
|---|---|---|
| WMS-98 03 | InChI=1S/C42H34F2NO3P.BrH/c43-34-24-18-31(19-25-34)42(41(45)47,32-20-26-35(44)27-21-32)33-22-28-36(29-23-33)48-40(46)17-10-30-49(37-11-4-1-5-12-37,38-13-6-2-7-14-38)39-15-8-3-9-16-39;/h1-9,11-16,18-29H,10,17,30H2,(H-,45,47);1H | Not extensively characterized yet (new mitochondria-targeted derivative of senicapoc; first data described in the manuscript) |
