# Supplementary material for: Metabolic Stress Shifts the Function of KCa3.1 Channels in Pancreatic Beta Cells From a Regulator of Membrane Potential to a Proapoptotic Factor
Source: Arch Pharm (Weinheim). 2026 Oct 9;359(10):e70344. doi: 10.1002/ardp.70344 (PMC13651915; doi:10.1002/ardp.70344)
Supplement: Supplementary file 2 [file ARDP-359-e70344-s002.docx]

**Supplementary Material**

**Metabolic stress shifts the function of K_Ca_3.1 channels in pancreatic beta cells from a regulator of membrane potential to a proapoptotic factor**

Jurek Hense^1^, Lena Beuke^1^, Christina Kick^2^, Bernhard Wünsch^2^ and Martina Düfer^1^*

^1^University of Münster, Institute of Pharmaceutical and Medicinal Chemistry, Dept. of Pharmacology, Corrensstraße 48, 48149 Münster, Germany

^2^University of Münster, Institute of Pharmaceutical and Medicinal Chemistry, Corrensstraße 48, 48149 Münster, Germany

*Correspondence:

Prof. Dr. Martina Düfer, University of Münster, Institute of Pharmaceutical and Medicinal Chemistry, Dept. of Pharmacology, Corrensstraße 48, 48149 Münster, Germany

Phone: (+49) 251 83 33339

Email: martina.duefer@uni-muenster.de

ORCID ID: 0000-0003-1572-9235

**Supplemental Figure 1:**

Influence of the L-type Ca_v_ channel inhibitor diltiazem on K^+^ currents of islet cells and lack of effects of senicapoc on K_ATP_ channels.

**Supplemental Figure 2:**

Ca^2+^-dependence of NMDAR-blockable K^+^ currents.

**Supplemental Figure 3:**

Whole-cell current-voltage relationships over a range from -60 to 80 mV.

**Supplemental Figure 4:**

Characterization of WMS-98 03.

**Supplemental Figure 5 and Table:**

Sex-specific data evaluation.

**Supplemental Figure 1**

Influence of the L-type Ca_v_ channel inhibitor diltiazem on K^+^ currents of islet cells and lack of effect of 1 µmol/L senicapoc on K_ATP_ channels. **(A)** Whole-cell current-voltage relationships were acquired in the presence of the L-Type Ca_V_ channel inhibitor diltiazem (1 µmol/L), a combination of diltiazem and senicapoc (1 µmol/L) or control conditions. Same protocol as illustrated in Figure 4D. **(B)** Whole-cell K_ATP_ current was measured by voltage-steps from the holding potential of -70 mV to -60 mV and -80 mV for 300 ms each. senicapoc (1 µmol/L) was applied after a stable K_ATP_ current had developed. The current amplitude between -70 and ‑60 mV was analysed. Tolbutamide (100 µmol/L) was added at the end of each experiment to verify that the current was current through K_ATP_ channels. * p < 0.05 for control *vs.* diltiazem, senicapoc

**Supplemental Figure 2**

Ca^2+^-dependence of NMDAR-blockable K^+^ currents. Whole-cell currents were measured at voltage steps to 0 mV for 300 ms from a holding potential of -70 mV. **(A)** Currents were measured before (black) and after (blue) the application of Ro25-6981 (1 µmol/L). Experiments were carried out in Ca^2+^ containing extracellular solution (left) and Ca^2+^ free extracellular solution to determine if the reduction of current elicited by Ro25-6981 is Ca^2+^ dependent. Ro25-6981 reduced the whole-cell current in Ca^2+^ containing extracellular solution in almost all cells, while it exerted no consistent effects in Ca^2+^ free solution resulting in no change of mean current. **(B)** Data are normalized to control without the NMDAR inhibitor. *** p < 0.001

**Supplemental Figure 3** Whole-cell current-voltage relationships over a range from -60 to 80 mV. **(A/B)** Complete I/V curves corresponding to data presented in Figure 1A and 1B. **(C/D)** Complete I/V curves corresponding to data presented in Figure 1C and 1D. **(E)** Complete I/V curve corresponding to data presented in Figure 2A.

**(A-D)** * p < 0.05, ** p< 0.01 for control vs. senicapoc; ## p < 0.01, ### p < 0.001 for control *vs.* nifedipine/Ro 25-6981; $ p < 0.05, $$ p < 0.01, $$$ p < 0.001 for senicapoc *vs.* nifedipine/Ro 25-6981; § p < 0.05 for nifedipine vs. nifedipine + senicapoc. **(E)** * p < 0.05 for NMDA/Gly *vs.* GLT + NMDA/Gly; # p < 0.05, ## p < 0.01, ### p < 0.001 for NMDA/Gly *vs.* NMDA/Gly + Ro25-698; $$$ p < 0.001 for GLT + NMDA/Gly *vs.* GLT + NMDA/Gly + Ro25-6981

**A**

HPLC Analysis of **WMS-98 03.**

**B**

^1^H NMR spectrum of **WMS-98 03** in DMSO-d_6_.

**C**

^13^C NMR spectrum of **WMS-98 03** in DMSO-d_6_.

**Supplemental Figure 4** Chromatogram **(A)** and NMR spectra **(B/C)** for WMS-98 03.

**A**

**B**

**C**

**-60**

**-40**

**-20**

**0**

**20**

**40**

**60**

**80**

**50**

**100**

**150**

**200**

**250**

**current density**

**(pA/pF)**

**voltage (mV)**

**female senicapoc (1 µmol/L) acute (n=23)**

**female control (n=22)**

**male senicapoc (1 µmol/L) acute (n=8)**

**male control (n=8)**

**control**

**-60**

**-40**

**-20**

**0**

**20**

**40**

**60**

**80**

**50**

**100**

**150**

**200**

**250**

**current density**

**(pA/pF)**

**voltage (mV)**

**female senicapoc (1 µmol/L) acute (n=23)**

**female control (n=23)**

**male senicapoc (1 µmol/L) acute (n=7)**

**male control (n=7)**

**GLT, 48 h**

**D**

**-60**

**-40**

**-20**

**0**

**20**

**40**

**60**

**80**

**50**

**100**

**150**

**200**

**250**

**current density**

**(pA/pF)**

**voltage (mV)**

**female Ro 25-6981, senicapoc acute (n=23)**

**female Ro 25-6981 (1 µmol/L) (n=22)**

**male Ro 25-6981, senicapoc acute (n=8)**

**male Ro 25-6981 (1 µmol/L) (n=8)**

**control**

**-60**

**-40**

**-20**

**0**

**20**

**40**

**60**

**80**

**50**

**100**

**150**

**200**

**250**

**current density**

**(pA/pF)**

**voltage (mV)**

**female Ro 25-6981, senicapoc acute (n=23)**

**female Ro 25-6981 (1 µmol/L) (n=23)**

**male Ro 25-6981, senicapoc acute (n=7)**

**male Ro 25-6981 (1 µmol/L) (n=7)**

**GLT, 48 h**

**A**

**B**

**C**

**-60**

**-40**

**-20**

**0**

**20**

**40**

**60**

**80**

**50**

**100**

**150**

**200**

**250**

**current density**

**(pA/pF)**

**voltage (mV)**

**female senicapoc (1 µmol/L) acute (n=23)**

**female control (n=22)**

**male senicapoc (1 µmol/L) acute (n=8)**

**male control (n=8)**

**control**

**-60**

**-40**

**-20**

**0**

**20**

**40**

**60**

**80**

**50**

**100**

**150**

**200**

**250**

**current density**

**(pA/pF)**

**voltage (mV)**

**female senicapoc (1 µmol/L) acute (n=23)**

**female control (n=23)**

**male senicapoc (1 µmol/L) acute (n=7)**

**male control (n=7)**

**GLT, 48 h**

**D**

**-60**

**-40**

**-20**

**0**

**20**

**40**

**60**

**80**

**50**

**100**

**150**

**200**

**250**

**current density**

**(pA/pF)**

**voltage (mV)**

**female Ro 25-6981, senicapoc acute (n=23)**

**female Ro 25-6981 acute (1 µmol/L) (n=22)**

**male Ro 25-6981, senicapoc acute (n=8)**

**male Ro 25-6981 (1 µmol/L) acute (n=8)**

**control**

**-60**

**-40**

**-20**

**0**

**20**

**40**

**60**

**80**

**50**

**100**

**150**

**200**

**250**

**current density**

**(pA/pF)**

**voltage (mV)**

**female Ro 25-6981, senicapoc acute (n=23)**

**female Ro 25-6981 acute (1 µmol/L) (n=23)**

**male Ro 25-6981, senicapoc acute (n=7)**

**male Ro 25-6981 acute (1 µmol/L) (n=7)**

**GLT, 48 h**

**-60**

**-40**

**-20**

**0**

**20**

**40**

**60**

**80**

**50**

**100**

**150**

**200**

**250**

**current density**

**(pA/pF)**

**voltage (mV)**

**female senicapoc (1 µmol/L) acute (n=23)**

**female control (n=22)**

**male senicapoc (1 µmol/L) acute (n=8)**

**male control (n=8)**

**A**

**B**

**-60**

**-40**

**-20**

**0**

**20**

**40**

**60**

**80**

**50**

**100**

**150**

**200**

**250**

**current density**

**(pA/pF)**

**voltage (mV)**

**C**

**female Ro 25-6981, senicapoc acute (n=23)**

**female Ro 25-6981 (1 µmol/L) (n=22)**

**male Ro 25-6981, senicapoc acute (n=8)**

**male Ro 25-6981 (1 µmol/L) (n=8)**

**-60**

**-40**

**-20**

**0**

**20**

**40**

**60**

**80**

**50**

**100**

**150**

**200**

**250**

**current density**

**(pA/pF)**

**voltage (mV)**

**female senicapoc (1 µmol/L) acute (n=23)**

**female control (n=23)**

**male senicapoc (1 µmol/L) acute (n=7)**

**male control (n=7)**

**control**

**GLT, 48 h**

**D**

**-60**

**-40**

**-20**

**0**

**20**

**40**

**60**

**80**

**50**

**100**

**150**

**200**

**250**

**current density**

**(pA/pF)**

**voltage (mV)**

**female Ro 25-6981, senicapoc acute (n=23)**

**female Ro 25-6981 (1 µmol/L) (n=23)**

**male Ro 25-6981, senicapoc acute (n=7)**

**male Ro 25-6981 (1 µmol/L) (n=7)**

**control**

**GLT, 48 h**

**Supplemental figure 5**

**Supplemental Figure 5:** Sex-specific evaluation of I/V curves. **(A)** The voltage protocol illustrated in Fig. 1G induces similar currents in beta cells of male and female mice after culture in standard medium in the absence or presence of senicapoc. **(B)** After 48-h culture in glucolipotoxic medium, beta-cells also behaved similarly under control conditions or in response to acute application of senicapoc. The influence of Ro 25-6981 +/- senicapoc after culture in standard **(C)** or glucolipotoxic medium **(D)** was similar in beta cells of male and female mice.

|  | male mice  mean±SD | female mice  mean±SD | male *vs.* female |
| --- | --- | --- | --- |
| K_slow_ control  15 mmol/L glucose acutely | 2.9±0.7 pA/pF,  n=11 beta-cells | 2.6±0.6 pA/pF,  n=7 beta-cells | n.s. |
| K_slow_ glucolipotoxicity (48 h)  15 mmol/L glucose acutely | 2.5±1.2 pA/pF,  n=11 beta-cells | 2.4±0.7 pA/pF,  n=7 beta-cells | n.s. |
| K_slow_ SUR1-KO control  15 mmol/L glucose acutely | 1.5±1.8 pA/pF,  n=19 beta-cells | 1.5±1.0, pA/pF,  n=9 beta-cells | n.s. |
| K_slow_ SUR1-KO  glucolipotoxicity (48 h)  15 mmol/L glucose acutely | 1.1±0.6, pA/pF,  n=18 beta-cells | 1.6±0.6 pA/pF,  n=8 beta-cells | n.s. |
| MEA glucolipotoxicity (48 h)  8 mmol/L glucose acutely | FOPP: 80±16 %,  n=5 islets | FOPP: 80±14,  n=5 islets | n.s. |
| MEA glucolipotoxicity (48 h)  8 mmol/L glucose acutely + 1 µM senicapoc | FOPP: 88±5 %,  n=5 islets | FOPP: 78±19 %,  n=5 islets | n.s. |

**Supplemental Table** Sex-specific analysis of K_slow_ currents and electrical activity. Separate statistical evaluation was only performed for data sets with a minimum of five experiments.

n.s. = not significant
